# Assignment of virus and antimicrobial resistance genes to microbial hosts in a complex microbial community by combined long-read assembly and proximity ligation

**DOI:** 10.1186/s13059-019-1760-x

**Published:** 2019-08-02

**Authors:** Derek M. Bickhart, Mick Watson, Sergey Koren, Kevin Panke-Buisse, Laura M. Cersosimo, Maximilian O. Press, Curtis P. Van Tassell, Jo Ann S. Van Kessel, Bradd J. Haley, Seon Woo Kim, Cheryl Heiner, Garret Suen, Kiranmayee Bakshy, Ivan Liachko, Shawn T. Sullivan, Phillip R. Myer, Jay Ghurye, Mihai Pop, Paul J. Weimer, Adam M. Phillippy, Timothy P. L. Smith

**Affiliations:** 1 Cell Wall Biology and Utilization Laboratory, Dairy Forage Research Center, USDA, Madison, WI 53706 USA; 2 Division of Genetics and Genomics, The Roslin Institute, Royal (Dick) School of Veterinary Studies, University of Edinburgh, Easter Bush, EH25 9RG UK; 3 Genome Informatics Section, Computational and Statistical Genomics Branch, National Human Genome Research Institute, Bethesda, MD USA; 4 Department of Animal Sciences, University of Florida, Gainesville, FL 32611 USA; 5 Phase Genomics Inc, Seattle, WA 98109 USA; 6 Animal Genomics and Improvement Laboratory, Beltsville Agricultural Research Center, Agricultural Research Service, USDA, Beltsville, MD 20705 USA; 7 Environmental Microbial and Food Safety Laboratory, Beltsville Agricultural Research Center, Agricultural Research Service, USDA, Beltsville, MD 20705 USA; 8 Pacific Biosciences, Menlo Park, CA USA; 9 Department of Bacteriology, University of Wisconsin-Madison, Madison, WI 53706 USA; 10 Department of Animal Science, University of Tennessee, Knoxville, TN 37996 USA; 11 Department of Computer Science, University of Maryland, College Park, MD 20742 USA; 12 USDA-ARS U.S. Meat Animal Research Center, Clay Center, NE 68933 USA

**Keywords:** Hi-C, Metagenomics, Virus-host association, PacBio, Metagenome assembly

## Abstract

We describe a method that adds long-read sequencing to a mix of technologies used to assemble a highly complex cattle rumen microbial community, and provide a comparison to short read-based methods. Long-read alignments and Hi-C linkage between contigs support the identification of 188 novel virus-host associations and the determination of phage life cycle states in the rumen microbial community. The long-read assembly also identifies 94 antimicrobial resistance genes, compared to only seven alleles in the short-read assembly. We demonstrate novel techniques that work synergistically to improve characterization of biological features in a highly complex rumen microbial community.

**Electronic supplementary material:**

The online version of this article (10.1186/s13059-019-1760-x) contains supplementary material, which is available to authorized users.

## Background

Microbial genome assembly from metagenomic sequence of complex communities produces large numbers of genome fragments, rather than complete circular genomes, despite continuous improvements in methodology [1, 2]. Assembly is complicated by sequences that may occur repeatedly within strains (“repeats”) or shared among similar strains of bacterial and archaeal species, creating “branches” in the assembly graph that precludes accurate representation of individual component genomes, particularly when multiple closely related strains of a species are present in the environment [3]. Repetitive content contributes to difficulty in multicellular Eukaryotic genome assembly as well [4], but the problem becomes more complicated in metagenome assembly [5] due to the wide range of abundance among bacterial species and strains, and the presence of other environmental DNA (e.g., plants, protists).

The application of long-read sequencing appears to be a potential solution to many of the difficulties inherent to metagenomic assembly. Read lengths that exceed the size of highly repetitive sequences, such as ribosomal RNA gene clusters, have been shown to improve contig lengths in the initial assembly [6, 7]. However, longer repetitive regions are only capable of being completely resolved by long reads of equal or greater size to the repeat, which makes input DNA quality a priority in sequence library construction. This can present a problem in metagenomic samples as material-adherent bacterial populations produce tough extracellular capsules that require vigorous mechanical stress for lysis, resulting in substantial DNA fragmentation and single-strand nicks [8]. Long-read sequencing technologies have been previously used in the assembly of the skin microbiome [9], in several environmental metagenomes [10], and in the binning of contigs from a biogas reactor [11]; however, each of these projects has relied on additional coverage from short-read data to compensate for lower long-read coverage. Additionally, higher depths of coverage of long reads from current generation sequencing technologies are necessary to overcome high, relative error rates that can impact assembly quality and influence functional genomic annotation [12]. Still, there is a substantial interest in generating assemblies derived from longer reads to enable better characterization of environmental and complex metagenomic communities [10]. Metagenome WGS assemblies consisting entirely of long reads have yet to be fully characterized, particularly those from complex, multi-kingdom symbiotic communities.

The bovine rumen is an organ that serves as the site of symbiosis between the cow and microbial species from all three taxonomic superkingdoms of life that are dedicated to the degradation of highly recalcitrant plant polymers [13]. With efficiency unrivaled by most abiotic industrial processes, the protists, archaea, bacteria, and fungi that make up the rumen microbial community are able to process cellulose and other plant biopolymers into byproducts, such as volatile fatty acids (VFA), that can be utilized by the host. This process is supplemented by relatively minimal energy inputs, such as the basal body temperature of the host cow and the energy-efficient mastication of digesting plant material. The presence of organisms from all major superkingdoms in varying degrees of abundance makes the rumen an excellent model for a complex, partially characterized metagenome system. Assessments of rumen microbial presence and abundance have generally been limited to 16S rRNA amplicon sequencing [14–16]; however, recent genome assemblies of metagenomic samples [17, 18] or isolates [19] derived from the rumen provide suitable standards for the comparison of new assembly methods and techniques.

In this study, we compare and contrast several different technologies that are suitable for metagenome assembly and binning, and we highlight distinct biological features that each technology is able to best resolve. We show that contigs generated using longer-read sequencing tend to be larger than those generated by shorter-read sequencing methods, long reads assemble more full-length genes and antimicrobial resistance gene alleles, and that long reads can be suitable for identifying the host specificity of assembled viruses/prophages in a metagenomic community. We also highlight novel virus-host associations and the potential horizontal transfer of antimicrobial resistance genes (ARG) in rumen microbial species using a combination of long reads and Hi-C intercontig link data. Our data suggests that future metagenomic surveys should include a combination of different sequencing and conformational capture technologies in order to fully assess the diversity and biological functionality of a sample.

## Results

### Sample extraction quality and de novo genome assemblies

**Fig. 1:**
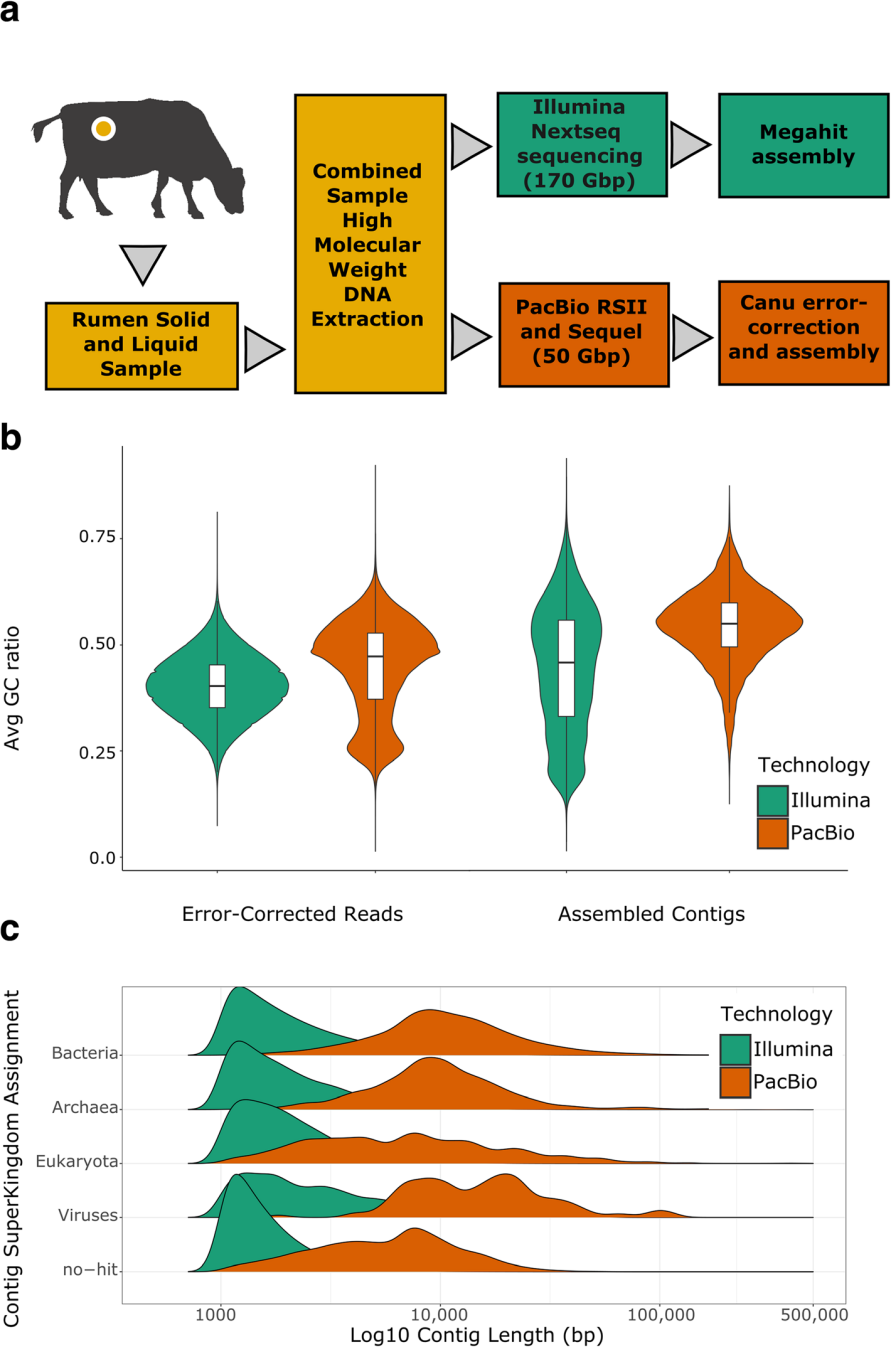
Assembly workflow and sampling bias estimates show GC% discrepancies in long-read vs short-read assemblies. Using the same sample from a cannulated cow, (**a**) we extracted DNA using a modified bead beating protocol that still preserved a large proportion of high molecular weight DNA strands. This DNA extraction was sequenced on a short-read sequencer (Illumina; dark green) and a long-read sequencer (PacBio RSII and Sequel; dark orange), with each sequence source assembled separately. Assessments of read- and contig-level GC% bias (**b**) revealed that a substantial proportion of sampled low GC DNA was not incorporated into either assembly. **c** Assembly contigs were annotated for likely superkingdoms of origin and were compared for overall contig lengths. The long-read assembly tended to have longer average contigs for each assembled superkingdom compared to the short-read assembly

We extracted high molecular weight DNA from a combined rumen fluid and solid sample taken from a single, multiparous, cannulated cow and sequenced that sample using a short-read and a long-read DNA sequencing technology (see the “Methods” section; Fig. 1a). The short-read and long-read data were assembled separately and generated de novo assemblies with contig N100K counts (the number of contigs with lengths greater than 100 kbp) of 88 and 384, respectively (Table 1). Both assemblies were generated with a minimum contig length cutoff of 1000 bp. While the short-read assembly contained fivefold more assembled bases (5.1 gigabases vs 1.0 gigabases), the long-read assembly was mostly comprised of larger contigs. We also observed a slight bias in the guanine-cytosine (GC) content of assembled contigs, with the short-read assembly having a larger sampling of different, average GC content tranches than the long-read assembly in observed, assembled contigs (Fig. 1b). Interestingly, the average GC content of the error-corrected long reads indicated a bimodal distribution at the 0.5 and 0.25 ratios (Fig. 1b) that is less pronounced in the GC statistics of the raw short reads and both sets of assembly contigs. There are several possibilities for this discrepancy; however, it is possible that this lower GC content range belongs to unassembled protist or anaerobic fungi genomes which are known to be highly repetitive and have low GC content [20, 21].

**Table 1:** Assembly statistics

Assembly	Contigs	Total assembly length (bp)	Contig N100K^1^
Illumina	2,182,263	5,111,042,186	88
PacBio	77,670	1,076,426,244	384

^1^The contig N100K is defined as the total number of contigs that are greater than 100 kbp in length in the entire assembly

We noticed a slight discrepancy in the superkingdom-specific contig lengths that suggests that many of our contigs of potential Eukaryotic origins are shorter than those of the Bacteria and Archaea, which coincided with our observation of GC content bias in the assembly (Fig. 1c). To assess the bias in GC content in our assembly of the long-read data, we calculated the overlap of raw long reads with our long-read assembly contigs. Density estimates of long reads that were not included in the long-read assembly (zero overlaps) mirrored the bimodal distribution of GC content in the raw long reads previously observed, suggesting that a larger proportion of lower GC content reads had insufficient coverage to be assembled (Additional file 1: Figure S1). Furthermore, we note that the error-corrected long reads were filtered based on intra-dataset overlaps, resulting in a further reduction of bases compared with the starting, raw long reads. The correction step removed 10% of the total reads for being singleton observations (zero overlaps with any other read) and trimmed the ends of 26% of the reads for having less than 2 overlaps. This may have also impacted the assembly of low abundance or highly complex genomes in the sample by removing rare observations of DNA sequence. We attempted to combine both the short-read and long-read datasets into a hybrid assembly; however, all attempts using currently available software were unsuccessful as currently available tools had prohibitive memory or runtime requirements due to the size of our input assemblies. We also investigated the use of long reads in multiple-datasource scaffolding programs and found only minor improvements in assembly size that were achieved through the inclusion of a high number of ambiguous base pairs (Additional file 1: Supplementary methods).

### Comparing binning performance and statistics

**Fig. 2:**
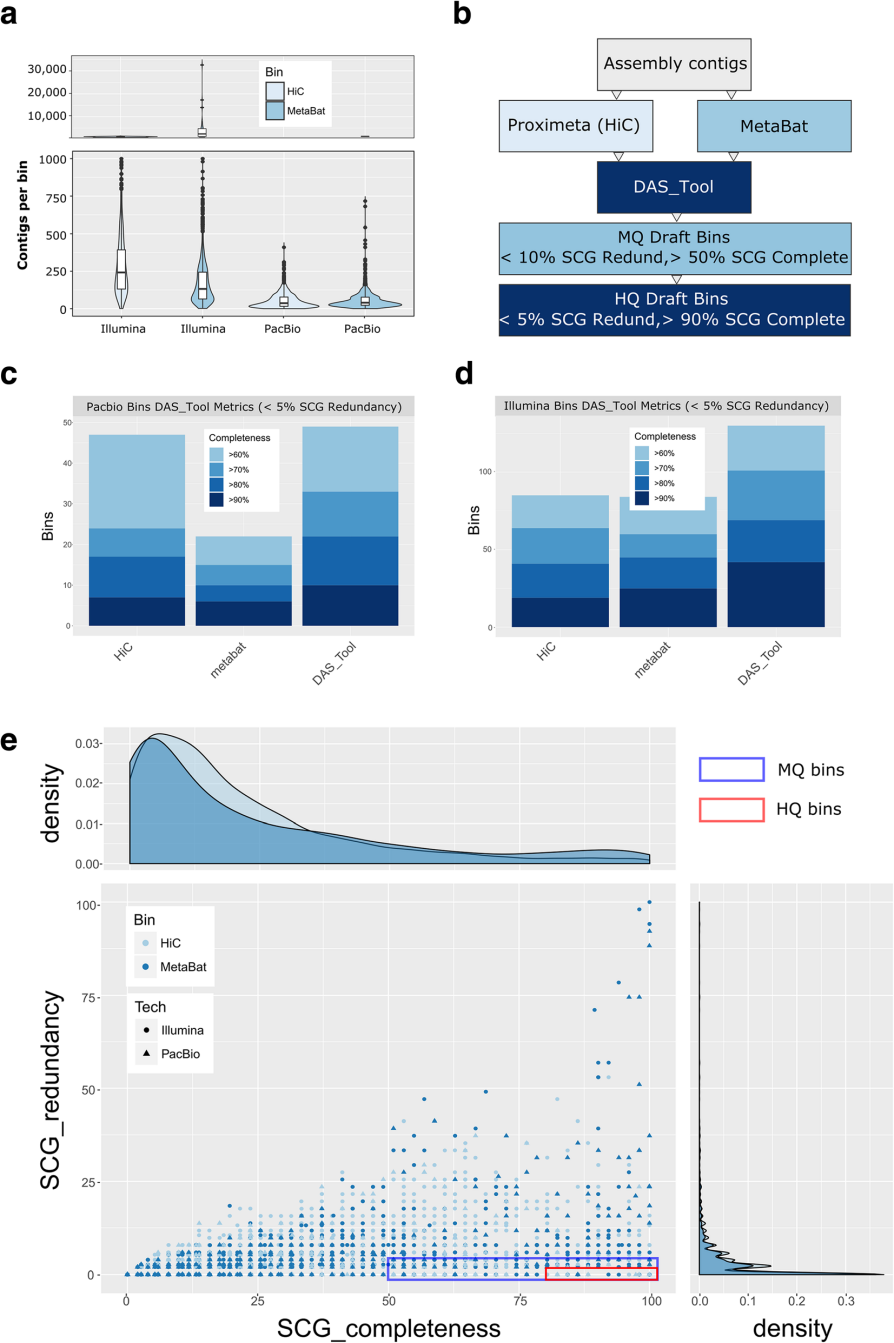
Identification of high-quality bins in comparative assemblies highlights the need for dereplication of different binning methods. **a** Binning performed by Metabat (light blue) and Proximeta Hi-C binning (Hi-C; blue) revealed that the long-read assembly consistently had fewer, longer contigs per bin than a short-read assembly. **b** Bin set division into medium-quality draft (MQ) and high-quality draft (HQ) bins was based on DAS_Tool single-copy gene (SCG) redundancy and completeness. Assessment of SCG completeness and redundancy revealed 10 and 42 high-quality bins in the long-read (**c**) and short-read (**d**) assemblies, respectively. The Proximeta Hi-C binning method performed better in terms of SCG metrics in the long-read assembly. **e** Plots of all of identified bins in the long-read (triangle) and short-read (circle) assemblies revealed a wide range of chimeric bins containing high SCG redundancy. Bins highlighted in the blue rectangle correspond to the MQ bins identified by the DAS_tool algorithm while the red rectangle corresponds to the HQ bin set

We applied computational (MetaBat) [22] and conformational capture methods (ProxiMeta Hi-C) [23] in order to bin assembled contigs into clusters that closely resembled the actual genomic content of unique species of rumen microbes (Additional file 1: Supplementary methods). The number of contigs per bin varied based on the binning method; however, the long-read assembly bins had nearly an order of magnitude fewer contigs per bin than the short-read assembly regardless of the method (Fig. 2a). We also saw a clear discrepancy between binning methods, with ProxiMeta preferably binning smaller (< 2,500 bp) contigs with higher GC (> 42%) than MetaBat (chi-squared test of independence *p* < 0.001; Additional file 1: Figure S2).

We further assessed bin quality and removed redundant contig-bin assignments between methods, using the single-copy gene (SCG) metrics of cluster contamination and completeness from the DAS_Tool [24] package (Fig. 2c, d; Additional files 2 and 3). We then sorted the revised DAS_Tool bins into a set of high-quality draft (HQ) bins and medium-quality draft (MQ) bins according to the standards of Bowers et al. [25] (Fig. 2b; Table 2). Since DAS_Tool assesses bin quality using bacterial and archaeal SCG metrics, we note that many Eukaryotic-origin bins are underrepresented in our filtered datasets. We also note a discrepancy in bin quality metrics between DAS_Tool dereplicated bins and assessments made with CheckM [26] (see the “Methods” section). Our HQ bin dataset contains 42 and 10 draft microbial genomes in the short-read and long-read datasets, respectively, with at least a 90% SCG completeness estimate and with less than 5% SCG redundancy (Fig. 2e; Additional files 4 and 5). We note that only 19 and 9 of our short-read and long-read HQ bins, respectively, meet the additional requirements of the presence of 16S, 23S, 5S and at least 18 tRNA genes per the Bowers et al. [25] standards (Additional files 4 and 5). The MQ binset contained 325 and 103 short-read and long-read consolidated bins, respectively.

**Table 2:** Assembly bin taxonomic assignment and gene content

			Assembled sequence taxonomic affiliation (kbp)^1^				
Assembly	Bin set	Avg # complete ORFs per contig^2^	Archaea	Bacteria	Eukaryota	Viruses	No-hits
Illumina	Unbinned	1.31	46,405	3,419,539	125,885	6287	1,019,041
	MQ	3.39	4543	393,630	3906	71	14,113
	HQ	7.66	1056	75,467	575	4	523
PacBio	Unbinned	14.6	10,686	854,468	7707	2290	26,804
	MQ	20.8	885	149,168	811	50	501
	HQ	48.2	1809	20,711	512	0	17

^1^superkingdom taxonomic affiliation was based on contig-level assignments derived from the BlobTools/DIAMOND workflow

^2^Complete ORFs were defined as Prodigal predictions that had a “partial” status of “00,” which indicates the presence of a start and stop codon for the ORF

### Taxonomic classification reveals assembly bias

**Fig. 3:**
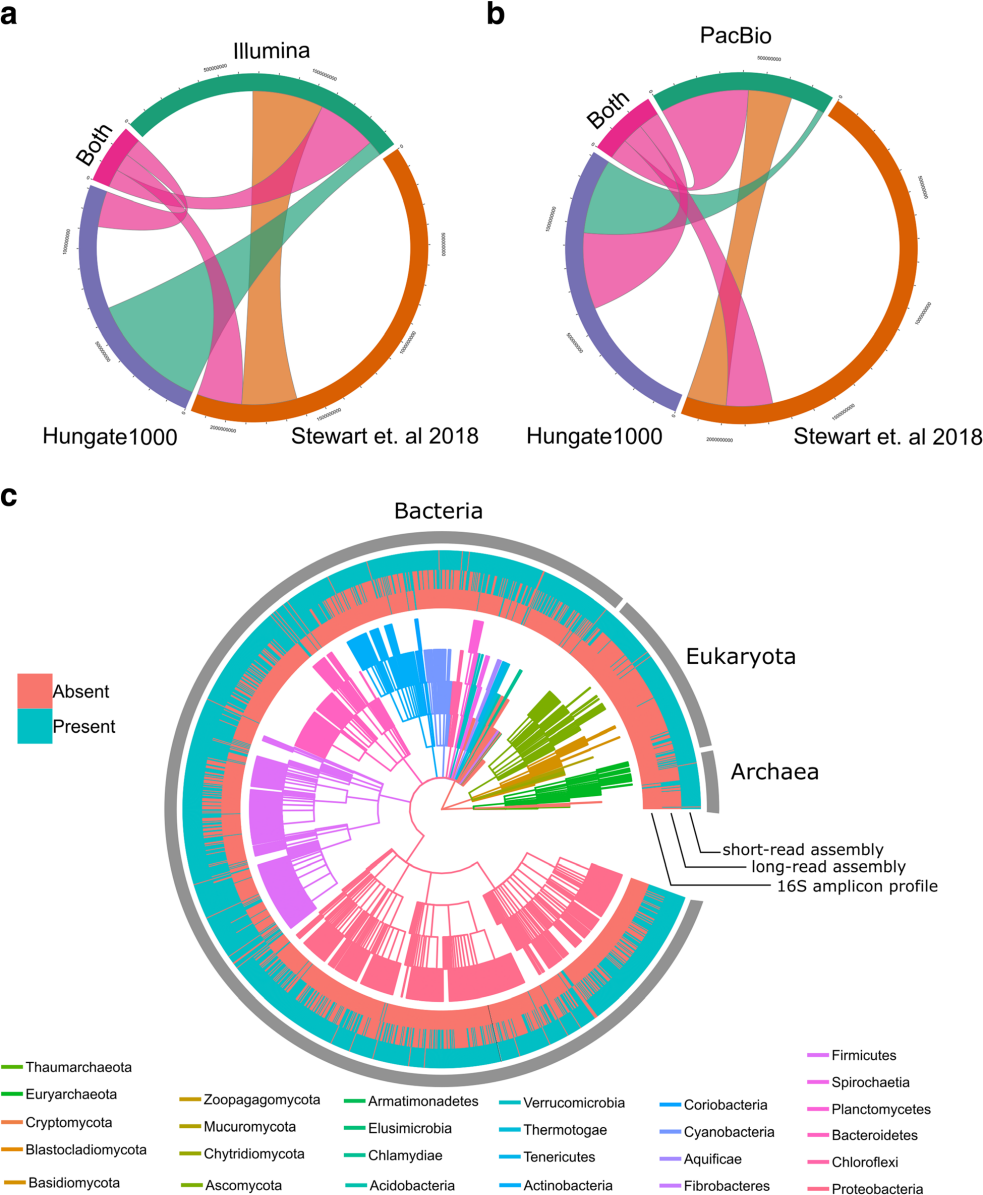
Dataset novelty compared to other rumen metagenome assemblies. Chord diagrams showing the contig alignment overlap (by base pair) of the short-read (**a**) and long-read (**b**) contigs to the Hungate1000 and Stewart et al. [18] rumen microbial assemblies. The “Both” category consists of alignments of the short-read and long-read contigs that have alignments to both Stewart et al. [18] and the Hungate1000 datasets. **c** A dendrogram comparison of dataset sampling completeness compared to 16S V4 amplicon sequence data analysis. The outer rings of the dendrogram indicate the presence (blue) or absence (red) of the particular phylotype in each dataset. Datasets are represented in the following order (from the outer edge to the internal edge): (1) the short-read assembly contigs, (2) the long-read assembly contigs, and (3) 16S V4 amplicon sequence data. The internal dendrogram represents each phylum in a different color (see legend), with individual tiers corresponding to the different levels of taxonomic affiliation. The outermost edge of the dendrogram consists of the genus-level affiliation

Taxonomic classification of the HQ bin and MQ binsets revealed a heavy preference towards the assembly of contigs of bacterial origin vs archaeal and eukaryotic origin (Fig. 3c; Additional file 1: Figure S3, S4), as expected from other surveys of the rumen [13]. Both the short- and long-read HQ bins each contain only one bin of archaeal-origin sequence. The short-read archaeal HQ bin was best classified as being a high-quality draft from the *Thermoplasmatales* order; however, the long-read archaeal bin was identified as belonging to the genus *Methanobrevibacter* from the family *Methanobacteriaceae*. Contig taxonomic assignment generated by the BlobTools [27] workflow varied greatly among the short-read HQ bins, with an average of 5 different phyla assignments per contig per bin compared to an average of 2.6 different assignments for the contigs in the long-read HQ bins (Additional files 6 and 7). We identified 14 full-length (> 1500 bp) predicted 16S rDNA genes in the long-read HQ bins, and only fragmentary (< 1500 bp) 16S genes in the short-read assembly (Additional file 8). The long-read MQ bins contained 64 full-length 16S genes, and all but 5 of the genes matched the original superkingdom taxonomic classification of the bin that contained the gene. Of these five discrepancies, four contigs were classified as “Eukaryotic” in origin, yet contained a predicted archaeal 16S gene.

### Comparison to other datasets reveals novel sequence

Contig novelty was assessed via direct overlap with other rumen metagenomic assemblies and via alignment with WGS reads from other publically accessible sources (Fig. 3a, b). We identified many contigs in our short-read and long-read assemblies that did not have analogous alignments to the recently published Stewart et al. [18] and Hungate 1000 [19] assemblies. From our HQ bins, 3650 and 22 contigs from the short- and long-read assemblies, respectively, did not align to any sequence in these two datasets, consisting of 25.4 Mbp and 317 kbp of assembled sequence that was missing from the previous, high quality, reference datasets for the rumen microbiome (Additional files 9 and 10). Expanding the comparison to the MQ binset, we identified 45,396 (179 Mbp) and 1254 contigs (16.1 Mbp) in the short- and long-read assemblies, respectively, that did not have analogs in the previous rumen datasets (Fig. 3a, b). From the MQ bins with no alignments to other published datasets, we identified 27,120 and 20 contigs in the short- and long-read MQ binsets, respectively, that did not have analogous alignments to the other respective dataset (e.g., short read vs long read). This represented 87.8 Mbp of the exclusive sequence in the short-read dataset not contained in our long-read dataset. However, we also identified 137 kbp that was novel to the long-read MQ bins despite the coverage disparity between the two datasets. Contigs that were exclusive to the long-read dataset were primarily of Firmicutes origin and had a higher median GC% value than other contigs in the long-read dataset (Kolmogorov-Smirnov *p* = 4.99 × 10^−4^). We wanted to compare the short-read sequence of our sample against other published rumen WGS datasets to see if there were differences in sample community composition that may have accounted for a novel assembled sequence in our dataset (Additional file 1: Supplementary methods; Table S2; Additional file 11). Our WGS reads were enriched for fungal and protist genomes compared to the selected public rumen WGS datasets (hypergeometric *p* value < 1 × 10^−7^ in all cases).

### Increased long-read contiguity results in more predicted ORFs per contig

We sought to assess whether the increased contiguity of the long-read assembly contigs provided tangible benefits in the annotation and classification of open reading frames (ORFs) in our MQ bin dataset. From Prodigal [28] annotation of the MQ bins from both assemblies, we identified 356,468 and 175,161 complete ORFs in the short-read and long-read assemblies, respectively (Additional files 12 and 13). We found a higher fraction of identified partial ORFs in the short-read MQ bins (142,434 partial; 28.5% of the complete ORF count) compared to the long-read MQ bins (9944 partial ORFs; 5.3% of the complete ORF count). This would suggest that, despite a lower total count of total ORFs identified, the long-read bins more frequently contained complete ORFs than did the short-read bins. We also found a higher mean count of ORFs per contig in the long-read MQ bins (mean 22.35) than the short-read bins (mean 3.75). This difference in average counts was found to be significant (Kolmogorov-Smirnov test *p* value < 0.001). In order to determine if this difference was due primarily to contig lengths, we divided all MQ bin contigs into quartiles by length and tested the average counts of complete ORFs in respective technology groups. We found only the bottom quartile (contig lengths less than 1705 bp) did not have significantly higher average counts after correction for multiple hypothesis testing (Kolmogorov-Smirnov test *p* = 0.022; Bonferroni-corrected *α* = 0.01); however, this may have been due to smaller sampling in the long-read dataset (only 17 contigs in this quartile) compared to the short-read dataset (20,555 contigs). All partial ORF predictions occur within the first and last 50 bp of contigs in the short-read and long-read MQ bins, suggesting that ORFs were prematurely terminated by contig breaks. In the short-read MQ bins, a surprising proportion of ORFs missing both a start and stop codon (4238 ORFs; 3.0% of the total count of partial ORFs) occur near the beginning of the contig compared to the long-read bin set (3 ORFs). However, we identified a slight discrepancy in ORF length between the short-read (median 758 bp) and long-read (median ORF length 653 bp) assemblies, with the former containing longer predicted ORFs than the long-read assembly. We did notice a small (linear model coefficient = 0.593), but significant (*F* test *p* value < 0.001), effect of the average short-read coverage of a contig on the length of predicted ORFs in the long-read assembly. We also observed a large reduction in median ORF lengths within 50 bp of the long-read contig ends (470-bp median length) compared to ORFs internal to the contig (668 bp), where short-read coverage was typically highest. This suggests that short-read coverage was still necessary to correct for some INDELs in the ORFs of the long-read assembly and that lower short-read coverage near the ends of contigs could have resulted in this discrepancy.

### Host-prophage association and CRISPR array identification

**Fig. 4:**
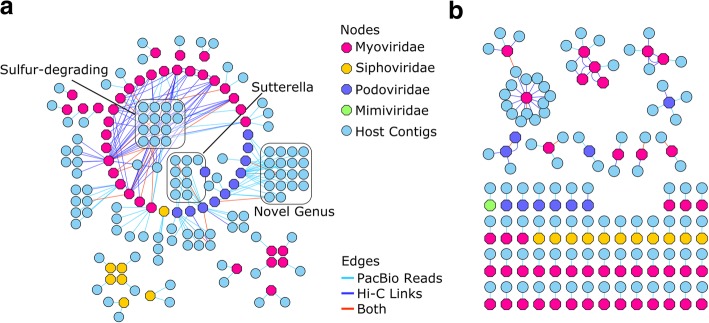
Network analysis of long-read alignments and Hi-C intercontig links identifies hosts for assembled viral contigs. In order to identify putative hosts for viral contigs, PacBio read alignments (light blue edges) and Hi-C intercontig link alignments (dark blue edges) were counted between viral contigs (hexagons) and non-viral contigs (circles) in the long-read assembly (**a**) and the short-read assembly (**b**). Instances where both PacBio reads and Hi-C intercontig links supported a virus-host assignment are also labeled (red edges). The long-read assembly enabled the detection of more virus-host associations in addition to several cases where viral contigs may display cross-species infectivity. We identified several viral contigs that infect important species in the rumen, including those from the genus *Sutterella*, and several species that metabolize sulfur. In addition, we identified a candidate viral association with a novel genus of rumen microbes identified in this study

Longer reads have the potential to provide direct sequence-level confirmation of prophage insertion into assembled genomes by spanning direct repeats that typically flank insertion sites [29]. To identify candidate host specificity for assembled prophage genomes, we used a heuristic alignment strategy with our error-corrected long-reads (Additional file 1: Supplementary methods) and Hi-C intercontig link density calculations. PacBio sequence data have a known propensity for chimerism [30]; however, we assumed that identical, chimeric PacBio reads would be unlikely to be seen more than once in our dataset. Similarly, we filtered Hi-C read alignments to identify virus-host contig pairs with higher link counts to identify virus-host associations in each assembly (Additional file 1: Supplementary Methods). Several viral contigs in the long-read assembly had substantial associations with contig groups affiliated with more than one genus (a maximum of 11 distinct genus-level classifications for one viral contig from the Myoviridae), suggesting a wide host specificity for these species (Fig. 4a). Long-read assembly viral contigs with multiple candidate host associations were identified as belonging to the Podoviridae, Myoviridae, and Siphoviridae families, which are viral families typically encountered in bovine rumen microbial samples [31]. Viral contigs from the short-read assembly were associated with fewer candidate host genus OTUs (four distinct associations at maximum; Fig. 4b). It is possible that the shorter length of Illumina assembly viral contigs (average size 4140 bp, standard deviation (sd) 5376 bp) compared with the long-read assembly contigs (average 20,178 bp, sd 19,334 bp) may have reduced the ability to identify host-phage associations in this case. Having identified read alignments between viral contigs and non-viral contigs, we sought to leverage conformational capture via Hi-C to see if we could confirm the virus-host associations.

We found that our Hi-C link analysis and PacBio read alignment analysis had very little overlap; however, we identified a tendency for each method to favor a different class of virus-host association which suggested that the methods were complementary rather than antagonistic (Additional file 14). Approximately 10% (short-read 6 out of 109; long-read 19 out of 188 pairs) of the host-viral contig associations had supporting evidence from both PacBio read alignments and Hi-C intercontig links. In nearly all highly connected viral contig pairs (greater than two additional contig associations), we observed evidence of host specificity from both methods even if it was for different host contigs. We also identified a bias in the virus-host family associations, where putative hosts for the Myoviridae were more likely to be identified via Hi-C than other viral families (Fig. 4a). Myoviridae family viral specificity for the sulfur-reducing *Desulfovibrio* and the sulfur-oxidizing *Sulfurovum* genera were primarily identified through Hi-C contig links (Fig. 4a, box: “Sulfur-degrading”). However, viral associations between the *Sutterella* and previously unreported genera of rumen bacteria were primarily identified via PacBio read alignments and had little Hi-C intercontig link support.

**Fig. 5:**
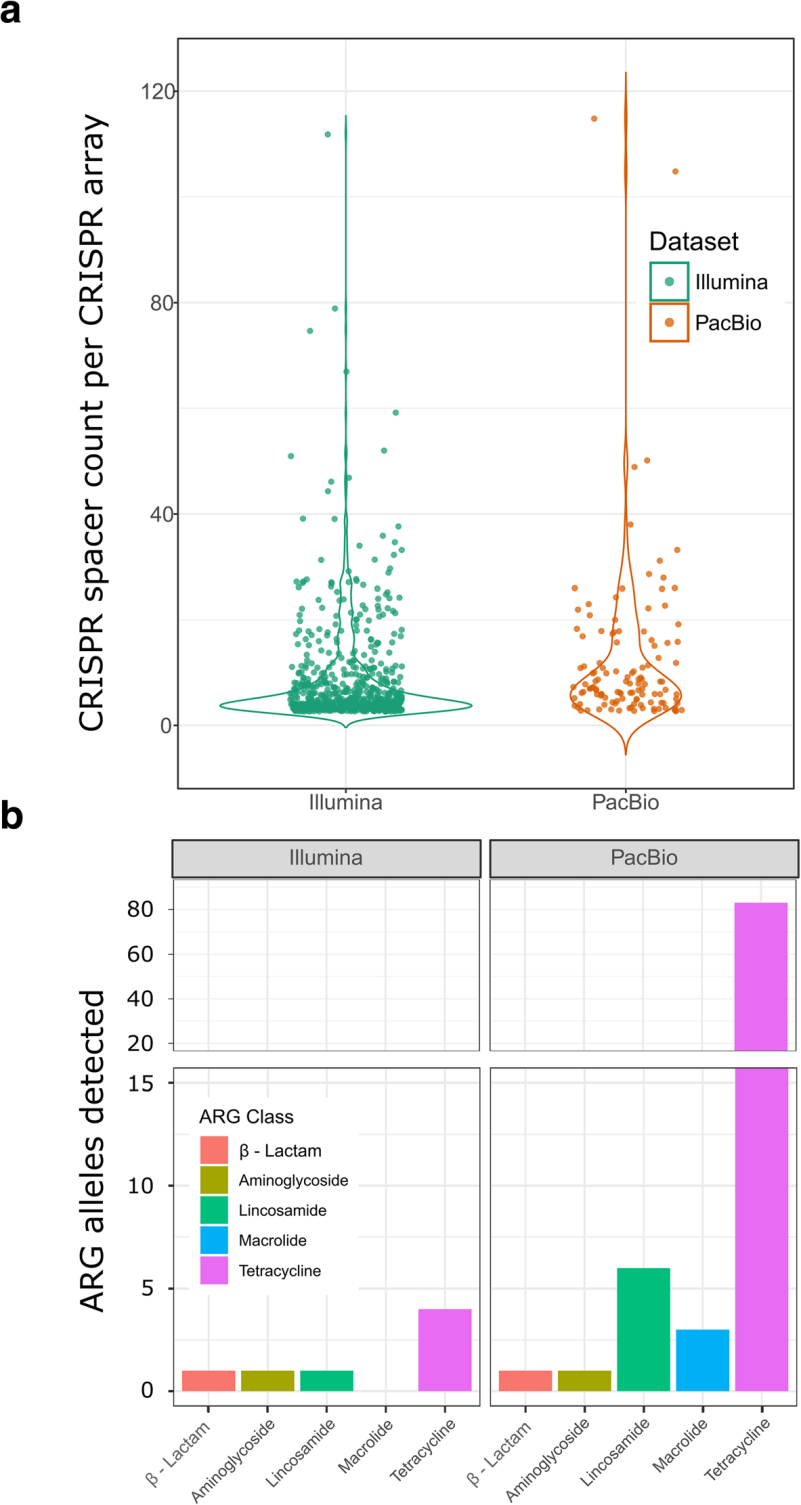
CRISPR array identification and ARG allele class counts were influenced by assembly quality. **a** The long-read assembly (dark orange) contigs had fewer identified CRISPR arrays than the short-read contigs (dark green); however, the CRISPR arrays with the largest count of spacers were overrepresented in the long-read assembly. **b** The long-read assembly had 13-fold higher antimicrobial resistance gene (ARG) alleles than the short-read assembly despite having 5-fold less sequence data coverage. The macrolide, lincosamide, and tetracycline ARG classes were particularly enriched in the long-read assembly compared to alleles identified in the short-read assembly

We also tested the ability of longer read sequence data to resolve highly repetitive bacterial defense system target motif arrays, such as those produced by the CRISPR-Cas system, in our dataset. Despite having less than one third of the coverage of the short-read dataset, our long-read assembly contained two of the three large CRISPR arrays (consisting of 105 and 115 spacers, respectively) in our combined assembly dataset (Fig. 5a). The short-read dataset (597 CRISPR arrays) contained approximately fivefold more identifiable CRISPR arrays than the long-read dataset (122 arrays), which is commensurate with the difference in the size of each assembly (5 Gbp vs 1 Gbp, respectively).

### Antimicrobial resistance gene detection

Due to the frequent use of antibiotics in livestock production systems to treat disease and improve production, we wanted to assess the utility of longer reads in detecting novel ARG alleles in assembled microbial genomes (Fig. 5b). The long-read assembly (ARG allele count 94) was found to contain over an order of magnitude more identifiable ARG alleles than the short-read assembly (ARG allele count 7), despite the major coverage discrepancies between the two datasets. The major contributor to this discrepancy was found in the tetracycline resistance gene class, as the long-read assembly contained 80 ribosomal protection and 3 efflux ARGs that are predicted to confer tetracycline resistance. Sequence similarity of ARG alleles in the long-read assembly followed a pattern consistent with ARG class, though we noted a cluster of *tetQ* and *tetW* alleles with less than 97% sequence similarity to other alleles of the same resistance class (Additional file 16). By contrast, a β-lactamase, lincosamide nucleotidyltransferase, and two tetracycline ARGs were identified in the short-read assembly and all four short-read ARGs had 99.02–100% sequence identity to equivalent ARG orthologs in the long-read assembly. Using the contigs containing these ARG alleles as anchors in our alignment of Hi-C read pairs, we attempted to identify horizontal transfer of these alleles using Hi-C intercontig link signal (Additional file 1: Supplementary Methods). We identified clusters of *Prevotella* bins and clusters of bins from the Clostridiales and Bacteroidales that higher contig link density with ARG allele contigs in our dataset (Additional file 1: Figure S5; Additional file 15). These associations may represent potential horizontal transfer of these alleles; however, we note that intercontig link density was relatively low in our comparisons (average alignments density was less than 2 reads per pair) and that ambiguous alignment to orthologous sequence could present false-positive signal in this analysis.

## Discussion

Whole metagenome shotgun sequencing and assembly has often relied exclusively on short-read technologies due to the cost-effectiveness of the methods and the higher throughput that they provide. While such strategies are often able to efficiently generate sufficient read depth coverage to assemble fragments of organisms in the community, we demonstrate that biases inherent in singular technologies suitable for metagenome assembly result in an incomplete or incorrect assembly/binning of the actual community. For example, we assembled a member of the archaeal order *Thermoplasmatales* in our short-read HQ bin dataset and a member of the archaeal genus *Methanobrevibacter* in the long-read HQ bins. Several taxonomic studies using short-read 16S-based methods have shown that the CO_2_-reducing *Methanobrevibacter* are one of the most abundant genera of methanogenic Archaea in the rumen [31], which was not reflected in our short-read HQ bins despite higher depths of coverage. Comparisons of both short- and long-read alignments suggest both Archaea are present in each respective dataset; however, errors incorporated in assembly and binning likely prevented an assembly or proper binning of the *Methanobrevibacter* genus in the short-read dataset. Conversely, we found that the short-read assembly contained more contigs assigned to the Eukaryotic superkingdom, which were relatively underrepresented in the long-read assembly. Given that we sequenced the same biological sample in all of our analyses, these discrepancies suggest that each technology samples different portions of the rumen microbial community. We acknowledge that differences in library preparation, DNA size fractionation, and other inherent biases in each technology prevent perfect comparisons between them. Additionally, comparisons of the content and composition of our short-read and long-read datasets must be tempered by the fact that they are sampling different depths (~ 170 Gbp vs ~ 50 Gbp, respectively) and fragments (~ 1133 million vs ~ 6 million reads) of the community. Still, our data suggest that each technology likely has a unique purview that can be attributed to compositional differences of the genomes among taxonomic superkingdoms (Fig. 1c), genomic GC% (Fig. 1b), and the presence of mobile DNA (Fig. 4, Additional file 1: Figure S6).

We identified a GC% bias in our short-read data relative to our long-read reads; however, this relative bias was reversed in comparisons of the GC content of the final assemblies, where our short-read assembly had more—albeit shorter—assembled contigs in lower GC% tranches (Fig. 1b). These differences are most likely due to the different error rates and degrees of coverage of reads from the two sequencing technologies and the algorithms used by the different assembly programs to correct for errors. Paradoxically, the short-read assembly sampled proportionally fewer reads at higher and lower GC tranches, but was able to incorporate even fragmentary information from these tranches into smaller contigs. The long-read assembly, by contrast, required sufficient coverage of reads to appropriately correct for errors and this meant that many lower GC% reads were discarded due to assembly constraints, as we demonstrate in our read alignment overlap analysis (Additional file 1: Figure S1). Protists may represent a large proportion of this lower GC% community, and their genomes likely consist of highly repetitive sequence that would require higher depths of long-read coverage to sufficiently traverse [21]. The use of improved error-correction methods or circular-consensus sequence reads [11, 32] is likely to provide substantial benefits for downstream annotation and may enable the assembly of the low-abundance, low-GC% species that were poorly represented in our long-read assembly. However, we acknowledge that size selection for longer fragments to sequence on our long-read dataset may have added additional bias. Comparisons of coverage between the two datasets on each respective assembly suggest that such bias may have a slight effect on sampled community composition (Additional file 1: Figure S6, S7; Supplementary Methods). This is a potential complication in using the long-read sequencing platform used in this study, as size selection is often required to improve subread N50 lengths.

We identified many biological features in our sample that would be missed if only a single technology/method was used for each step of the assembly, binning and analysis of our dataset. Larger contigs in the long-read dataset also resulted in a higher average count of annotated ORFs per contig than the short-read dataset by a factor of seven. This contiguity of gene regions is particularly important in bacterial classification, where functional genes of particular classes can be arranged in complete and phased operons. It is highly likely that this increase in contiguity contributed to the massive discrepancy in ARG allele identification between the two assemblies; however, we also note that the high percent identity of ARG allele orthologs may have contributed to this issue. Similar to how longer reads are able to resolve large repetitive clusters in Eukaryotic genome assembly [6, 7], reads that are longer than the highly repetitive ARG alleles may have resulted in increased detection in the long-read assembly, whereas the short-read assembly would have generated a contig break. We noted a significant increase in detected tetracycline resistance alleles in our long-read assembly of a rumen metagenome from a concentrate-fed animal, which contradicts previous work using short-read assemblies that found that animals fed concentrates should have few tetracycline resistance alleles [33]. Calves in the sampled research herd (UW-Madison, Dairy Forage Research Center) are given chlortetracycline during inclement weather and tetracycline is applied topically to heel warts on adult animals. It is possible that incidental/early exposure to this antibiotic has enabled the proliferation of tetracycline resistance alleles in the rumen community, and this proliferation was only detected in our long-read assembly. Previous studies have demonstrated the benefit of using longer reads in ARG allele-associated satellite DNA tracking [34] and ARG allele amplicon sequencing [35]. To our knowledge, this is the first survey to identify the benefits of long reads in de novo assembly of ARG alleles from a complex metagenomic sample.

We also identified discrepancies between our selected computational (MetaBat) and proximity ligation (ProxiMeta Hi-C) binning methods that suggest that a combination of binning techniques is needed to identify all complete MAGs in a metagenomic sample. We note that Hi-C linkage data is dependent on the density of selected restriction sites in the genomes of the community and the protein-DNA interactions that are selectively enriched during library preparation (Additional file 1: Supplementary methods). This difference in sampling composition from our short-read WGS read dataset means that it is difficult to distinguish between the biases of each method and real biological signal, so our comparisons are limited to the observed content of bins from each technology on the same dataset. Results from the short-read and long-read assemblies are concordant, which suggests that the general output of the binning programs is agnostic to the sequencing technology in our dataset. Contig binning comparisons suggest that MetaBat successfully binned contigs from the low-GC% contig tranches; however, it failed to incorporate the same proportion of smaller contigs in bins from the short-read (< 2500 bp) or long-read (< 10,000 bp) assemblies as the ProxiMeta method. Smaller contigs most likely result from low-sequencing coverage regions or high copy orthologous genomic segments in a metagenomic sample. Both of these problems may have confounded the tetranucleotide frequency and coverage depth estimates used by MetaBat to bin our contigs, resulting in their lower frequencies in that binset. We did note some issues in DAS_tool dereplication of our dataset, where DAS_tool may have aggressively pruned contigs from MetaBat bins. However, our data suggests that MetaBat may have included far more contamination due to cross-Kingdom SCGs, thereby resulting in this aggressive filtration (for more details, please see the “Genome assembly and binning” section of the “Methods” section).

In order to identify the horizontal transfer of mobile DNA in the rumen, we exploited two technologies to identify candidate hosts for transferred ARG alleles and assembled viral contigs. We observed intercontig link associations between ARG allele contigs and bins that consisted of species from the Clostridiales and Bacteroidales. Evidence of identical ARG allele orthologs belonging to both classes was previously found in human colon samples [36]; however, we note that our analysis shows only a precursory association of the context of identified ARG alleles and prospective host bins. We were unable to identify the exact vector that may enable the cross-species transfer of several of these alleles, but we suspect that lateral transfer of ARG alleles may be an adaptation of rumen bacterial species against antibiotic challenge as noted above. Direct evidence of the horizontal transfer of mobile elements was observed in identified novel virus-host associations that we detected by using a combination of PacBio long-read alignments and Hi-C intercontig link analysis. Proximity ligation has been previously used to detect virus-host associations [37]; however, our combination of technologies potentially reveals new insights in the biology of the interaction between host and phage. We found a clear preference between the two methods in the detection of viral family classes, with Hi-C intercontig links preferring the Myoviridae viral family and our PacBio read alignments preferring all other viral families. This preference may reflect the nature of the activity of these viruses, as some genera of the Myoviridae family are known to have short lytic cycles [38] as opposed to long-term lysogenic life cycles found in other viral families. We also identified virus-host association with several contigs within bins identified as belonging to the *Desulfovibrio* and *Sulfurovum* genera. Viral auxiliary metabolic genes related to sulfur metabolism were previously identified in the assembly of rumen viral populations [39], and our study may provide a link to the putative origins of these auxiliary genes in host genomes that are known to metabolize sulfur compounds. We identified two ORFs annotated as 3′-phosphoadenosine-5′-phosphosulfate (PAPS) genes in a viral contig in the long-read assembly that was associated with host contigs assigned to the *Dehalococcoides*. We did not detect any auxiliary metabolic genes in the short-read assembly. Additionally, the short-read assembly served as the basis of fewer virus-host contig associations in both Hi-C and PacBio read analyses, suggesting that assembled short-read viral contigs may have been too small or redundant to provide a useful foundation for alignment-based associations.

We recommend that future surveys of complex metagenomic communities include a combination of different DNA sequencing technologies and conformational capture techniques (i.e., Hi-C) in order to best resolve the unique biological features of the community. If our analysis was restricted to the use of the short-read WGS data and one computational binning technique (MetaBat), we would have missed 139 out of 250 of the top dereplicated DAS_Tool short-read bins contributed by ProxiMeta binning. Our long-read dataset further contributed 7886 complete ORFS, 97 ARG alleles, and 188 virus-host associations, with Hi-C signal providing further evidence of virus-host associations. We demonstrate that even a small proportion of long-reads can contribute high-quality metagenome bins and that the long-read data provided by the technology is suitable for uncovering candidate mobile DNA in the sample. We also note that the inclusion of a computational binning method (Metabat) with a physical binning technique (ProxiMeta; Hi-C) further increased our count of high-quality, DAS_Tool dereplicated bins, likely due to each method sampling a different pool of organisms. Therefore, the DAS_Tool dereplication of both sets of bins increased our final counts of high-quality (> 80% completion) bins by 30–60% in the long-read and short-read assemblies. If a metagenomic WGS survey is cost-constrained, our data suggests that a computational method, such as MetaBat, currently cannot fully compensate for the sampling bias and repetitive, orthologous DNA issues that could reduce the completeness of a downstream short-read assembly. Still, we suspect that such projects will be able to assemble and characterize the abundant, moderate-GC portion of the metagenome community sufficiently for analysis.

Further refinements could improve characterization of the rumen microbial community and other complex metagenomic communities in general. We note that the majority of our HQ bins are already present in other rumen metagenome assemblies, suggesting that the highly abundant, “core” bacterial community has been sufficiently assembled in other surveys [18, 19]. However, microbes present in low abundance (or transient species) still represent a challenge to all of the technologies used in our survey. A sample fractionation method similar to one used by Solden et al. [40] would enable better, targeted coverage of these communities in future surveys while losing the ability to determine relative abundance estimates for strains. In the absence of targeted sample enrichment, co-assembly with other sampled datasets [18], low-error rate long reads [32], or real-time, selective read sequencing [41] would enable sampling of lower abundant strains. Additionally, there is a need for a rigorous method to combine and/or scaffold metagenome assemblies with high-error long reads. Our attempts to combine our short-read and long-read datasets using existing scaffolding and assembly software failed to produce a significant improvement in assembly contiguity and quality. The complexity of the data will likely require a specialized solution that can also resolve issues that result from excessive strain heterogeneity.

## Conclusions

We demonstrate the benefits of using multiple sequencing technologies and proximity ligation in identifying unique biological facets of the cattle rumen metagenome, and we present data that suggests that each has a unique niche in downstream analysis. Our comparison identified biases in the sampling of different portions of the community by each sequencing technology, suggesting that a single DNA sequencing technology is insufficient to characterize complex metagenomic samples. Using a combination of long-read alignments and proximity ligation, we identified putative hosts for assembled bacteriophage at a resolution previously unreported in other rumen surveys. These host-phage assignments support previous work that revealed increased viral predation of sulfur-metabolizing bacterial species; however, we were able to provide a higher resolution of this association, identify potential auxiliary metabolic genes related to sulfur metabolism, and identify phage that may target a diverse range of different bacterial species. Furthermore, we found evidence to support that these viruses have a lytic life cycle due to a higher proportion of Hi-C intercontig link association data in our analysis. Finally, it appears that there may be a high degree of mobile DNA that was heretofore uncharacterized in the rumen and that this mobile DNA may be shuttling antimicrobial resistance gene alleles among distantly related species. These unique characteristics of the rumen microbial community would be difficult to detect without the use of several different methods and techniques that we have refined in this study, and we recommend that future surveys incorporate these techniques to further characterize complex metagenomic communities.

## Methods

### Sample selection, DNA extraction, and Hi-C library preparation

Rumen contents from one multiparous Holstein cow housed at the University of Wisconsin, Madison, campus were sampled via rumen cannula as previously described [42] under a registered Institutional Animal Care and Use Committee protocol: A005902. The sampled cow was in a later period of lactation and was being fed a total mixed ration. Rumen solids and liquids were combined in a 1:1 volume mix, and then were agitated using a blender with carbon dioxide gas infusion as previously described [42]. DNA was extracted via the protocols of Yu and Morrison [43] albeit with several modifications to the protocol to increase yield. To improve DNA precipitation, an increased volume of 10 M ammonium acetate (20% of the supernatant volume) was added. Additionally, DNA pellets were not vacuum dried so as to reduce the potential for single-strand nicking due to dehydration. DNA quality was assessed via Fragment Analyzer spectra and spectrophotometric assays.

Portions of the rumen content samples were fixed by a low concentration formaldehyde solution before DNA extraction as previously described [44]. Fixed samples were subject to the same DNA extraction protocol as listed above, processed by Phase Genomics (Seattle, WA) and sequenced on a HiSeq 2000.

### Long-read and short-read DNA sequencing

Tru-seq libraries were created from whole DNA preps for the sample as previously described [45]. Samples were run on a single Illumina NextSeq500 flowcell using a 300 cycle SBS kit to produce 1.14 billion, 150 bp by 150 bp paired-end reads. The total amount of sequenced bases for the short-read dataset was 171 Gbp (Additional file 1: Table S1). Hi-C libraries were created as previously described [44], and sequenced on an Illumina Hiseq 2000 to generate 80 × 80 paired-end reads. A total of 40,889,499 and 22,487,509 reads for the Sau3AI and MluCI libraries were generated, respectively.

DNA samples from each cow were size selected to a 6-kb fragment length cutoff using a Blue Pippen (Sage Science; Beverly, MA). Libraries for SMRT sequencing were created as previously described [6] from the size-selected DNA samples. We generated 6.7 and 45.35 Gbp of PacBio uncorrected reads using the PacBio RSII (8 cells) and PacBio Sequel (21 cells), respectively. Different DNA extraction methods can result in substantial observed differences in strain- and species-level assignments depending on the recalcitrance of the cell wall of individual cells [8]. However, contemporary long-read sequencing platforms require input DNA to be devoid of single-strand nicks in order to maximize sequence read lengths [46]. Indeed, our observed, average subread length for the long-read dataset was almost half (7823 bp RSII; 6449 bp Sequel) the size of our original Fragment Analyzer spectra peaks (~ 14,651 bp), suggesting that the bacterial cell lysis still impacted DNA molecule integrity (Additional file 1: Figure S8). Regardless, a total of 52 Gbp of subread bases were generated on all samples using PacBio sequencers (Additional file 1: Table S1).

### Genome assembly and binning

PacBio raw reads were assembled by Canu v1.6+101 changes (r8513). We ran five rounds of correction to try to recover lower-coverage reads for assembly using the parameters “-correct corMinCoverage=0 genomeSize=5m corOutCoverage=all corMhapSensitivity=high”. The input for each subsequent round was the corrected reads from the previous step. Finally, the assembly was generated via the parameters “-trim-assemble genomeSize=5m oeaMemory=32 redMemory=32 correctedErrorRate=0.035”. The assembly was successively polished twice with Illumina data using Pilon restricted to fix indel errors using the “-fix indels” and “-nostrays” parameters. Pilon correction was automated using the slurmPilonCorrectionPipeline.py script available at the following repository: https://github.com/njdbickhart/RumenLongReadASM. We generated a second set of PacBio corrected reads for the viral association and GC-read overlap analyses using the options “-correct corMinCoverage=0 genomeSize=5m corOutCoverage=all corMhapSensitivity=high corMaxEvidenceCoverageLocal=10 corMaxEvidenceCoverageGlobal=10” to restrict the global filter to avoid over-smashing similar sequences during correction. Illumina reads were assembled using MegaHit v1.1.2 using parameters --continue --kmin-1pass -m 15e+10 --presets meta-large --min-contig-len 1000 -t 16 and otherwise default settings.

Reads from other rumen WGS datasets (Additional file 1: Table S2) were aligned to assembled contigs from both assemblies with BWA MEM [47] and were used in Metabat2 binning [22]. Metabat2 was run with default settings using the coverage estimates from all rumen WGS datasets (Additional file 1: Supplementary methods). Hi-C reads were aligned to assembled contigs from both assemblies using BWA MEM [47] with options -5S, and contigs were clustered using these alignments in the Phase Genomics ProxiMeta analysis suite [44]. We noted a difference in bin contamination between the two methods, where Metabat tended to have more bins with greater than 10% CheckM [26] Contamination (76 out of 1347 short-read bins) compared to the ProxiMeta bins (29 out of 3664 bins; chi-squared *p* < 0.001). We also briefly assessed the utility of Hi-C links against the use of short-read WGS, PE links on our dataset using the mmgenome2 R package [48] (Additional file 1: Figure S9, S10; Additional file 1: Supplementary methods). The quality of Hi-C library preparation was assessed by the proximity of read alignments to the motifs of each respective restriction endonuclease used to fragment the library (Additional file 1: Figure S11).

Using the ProxiMeta and MetaBat bin assignments as a seed, we consolidated assembly bins for each assembly using the DAS_Tool pipeline [24]. The dereplication algorithm of DAS_Tool modifies input bin composition in an iterative, but deterministic, fashion, so we also validated the quality of our input bins by using CheckM [26] quality metrics in addition to the DAS_Tool SCG metrics (Fig. 2c, d). We noted some discrepancies in the CheckM quality metrics and those estimated by DAS_Tool for our input and dereplicated MetaBat bins, respectively (Additional file 1: Figure S13, S14). CheckM tended to overestimate the quality of MetaBat input bins and dereplicated bins in each assembly, which may have due to the inclusion of proportionally more cross-Kingdom SCGs in the MetaBat bins as assessed by DAS_Tool. As a result, DAS_Tool dereplication was far more permissive at removing bins from our MetaBat dataset (average 69 ± 204 contigs removed per bin) than our ProxiMeta dataset (average 23 ± 30 contigs) in our short-read dataset. For further details on assembly binning and bin dereplication, please see Additional file 1: Supplementary methods. Finally, we assessed the proportion of short-read WGS reads that aligned to the bins that were generated by DAS_tool and found that the HQ bins comprised ~ 1.2% of the total short-read WGS alignments (Additional file 1: Figure S12).

### Assembly statistics and contaminant identification

General contig classification and dataset statistics were assessed using the Blobtools pipeline [27]. To generate read coverage data for contig classification, paired-end short-read datasets from 16 SRA datasets and the Illumina sequence data from this study were aligned to each contig and used in subsequent binning and contaminant identification screens. For a full list of datasets and accessions used in the cross-genome comparison alignments, please see Additional file 1: Table S2. Assembly coverage and contig classifications were visually inspected using Blobtools [27]. Comparisons between assembled contigs and other cattle-associated WGS metagenomic datasets were performed by using MASH [49] sketch profile operations and minimap2 [50] alignments. Datasets were sketched in MASH by using a kmer size (-k) of 21 with a sketch size of 10,000 (-s). Minmap2 alignments were performed using the “asm5” preset configuration. DIAMOND [51] alignment using the Uniprot reference proteomes database (release: 2017_07) was used to identify potential taxonomic affiliation of contigs through the Blobtools metagenome analysis workflow [27]. MAGpy [52] was also used to suggest putative names for the short- and long-read bins. CheckM [26] version 1.0.11 was used to assess bin contamination and completeness separately from the DAS_Tool SCG quality metrics.

### ORF prediction, gene annotation, and taxonomic affiliation

Open reading frames were identified by Prodigal [28] (v 2.6.3) as part of the DAS_Tool pipeline. Gene ontology (GO) term assignment was performed using the Eggnog-mapper pipeline [53] using the same DIAMOND input alignments used in the Blobtools analysis. Assembly bin functional classification was determined using the FAPROTAX workflow [54], using the Uniprot/DIAMOND/Blobtools-derived taxonomy of each contig. In order to deal with uncertain species-level classifications for previously unassembled strains, taxonomic affiliations were agglomerated at the genus level for dendrogram construction. The reference tree was created from NCBI Common Tree (https://www.ncbi.nlm.nih.gov/Taxonomy/CommonTree/wwwcmt.cgi) and plotted in the R package ggtree [55].

### Virus-host association prediction and Hi-C intercontig link analysis

In order to identify potential virus-host links, we used a direct long-read alignment strategy (PacBio alignment) and a Hi-C intercontig link analysis (Hi-C). Briefly, contigs identified as being primarily viral in origin from the Blobtools workflow were isolated from the short-read and long-read assemblies. These contigs were then used as the references in an alignment of the error-corrected PacBio reads generated in our second round of Canu correction (please see the “Genome assembly and binning” section above). We used Minimap2 to align the PacBio dataset to the viral contigs from both datasets using the “map-pb” alignment preset. Resulting alignment files (“paf”) were subsequently filtered using the “selectLikelyViralOverhangs.pl” script, to selectively identify PacBio read alignments that extend beyond the contig’s borders. We then used the trimmed, unaligned portions of these reads in a second alignment to the entire assembly to identify putative host contigs (Additional file 1: Supplementary methods). A virus-host contig pair was only identified if two or more separate reads aligned to the same viral/non-viral contig pair in any orientation.

Hi-C intercontig link associations were identified from read alignments of the Hi-C data to each respective assembly. BAM files generated from BWA alignments of Hi-C reads to the assemblies were reduced to a bipartite, undirected graph of intercontig alignment counts. The graph was filtered to identify only intercontig links that involved viral contigs and that had greater than 20 or 10 observations in the long-read and short-read assembly, respectively. The information from both methods was combined in a qualitative fashion using custom scripts (Additional file 1: Supplementary methods). The resulting dataset was visualized using Cytoscape [56] with the default layout settings, or the “attribute circle” layout option depending on the degrees of viral-contig associations that needed to be visually represented.

### CRISPR-CAS spacer detection and ARG detection

ARG homologues were identified using BLASTN with the nucleotide sequences extracted from the Prodigal ORF locations as a query against the transferrable ARG ResFinder database [57]. Hits with a minimum 95% nucleotide sequence identity and 90% ARG sequence coverage were retained as candidate ARGs. Hi-C linker analysis identifying ARG gene contig associations was derived from Proximeta bin data and Hi-C read alignments by counting the number of read pairs connecting contigs in each bin to each ARG. The procedure for identifying these associations was similar to the protocol used to identify Hi-C-based, virus-host associations. Briefly, a bipartite, undirected graph of intercontig alignment counts was filtered to contain only associations originating from contigs that contained ARG alleles and had hits to non-ARG-containing contigs. This graph was then converted into a matrix of raw association counts, which were then analyzed using the R statistical language (version 3.4.4). Taxonomic affiliations of contigs were derived from Blobtools, whereas the taxonomic affiliations of AN bins were derived from ProxiMeta MASH [49] and CheckM [26] analysis.

## Additional files

Additional file 1: Supplementary figures and methods. Contains all supplementary figures and two supplementary tables. Additionally, contains a brief listing of additional methods and command line code for replicating analysis. (DOCX 3839 kb)

Additional file 2: Short-read assembly bins. A tab-delimited text file containing contig ID names, contig lengths, bin assignment, and read depth coverage for the short-read assembly MQ bins. (GZ 13035 kb)

Additional file 3: Long-read assembly bins. A tab-delimited text file containing contig ID names, contig lengths, bin assignment, and read depth coverage for the long-read assembly MQ bins. (GZ 731 kb)

Additional file 4: Short-read assembly HQ bins. A tab-delimited text file listing the number of contigs, total sizes, and summary statistics for short-read assembly HQ bins. (TAB 4 kb)

Additional file 5: Long-read assembly HQ bins. A tab-delimited text file listing the number of contigs, total sizes, and summary statistics for long-read assembly HQ bins. (TAB 1 kb)

Additional file 6: Short-read assembly MQ bin taxonomy. A tab-delimited text file that lists the taxonomic assignment of short-read assembly MQ bins as determined by the Blobtools/DIAMOND alignment pipeline. (GZ 49157 kb)

Additional file 7: Long-read assembly MQ bin taxonomy. A tab-delimited text file that lists the taxonomic assignment of long-read assembly MQ bins as determined by the Blobtools/DIAMOND alignment pipeline. (GZ 6453 kb)

Additional file 8: 16S small subunit alignments in HQ bins. Identification and summary statistics on identified 16S fragment/full-length sequences in the HQ dataset. (XLSX 15 kb)

Additional file 9: Short-read unique rumen assembly sequence. A listing of the short-read contigs that did not have reciprocal alignments to the Hungate1000 or Stewart et al. rumen microbial assemblies. (GZ 17072 kb)

Additional file 10: Long-read unique rumen assembly sequence. A listing of the long-read contigs that did not have reciprocal alignments to the Hungate1000 or Stewart et al. rumen microbial assemblies. (GZ 9 kb)

Additional file 11: Hypergeometric test of contig alignment depth. These are the results of an enrichment test designed to identify differences in community abundance/composition between several public rumen datasets (see Additional file 1: Supplementary methods). The short-read assembly and long-read assembly results are listed on separate tabs. Enrichment was determined by the Hypergeometric mean test using a Benjamini-Hochberg-corrected alpha. (XLSX 224 kb)

Additional file 12: Short-read assembly Prodigal ORF predictions. This file contains all Prodigal ORF predictions for the short-read MQ bins. (GZ 87178 kb)

Additional file 13: Long-read assembly Prodigal ORF predictions. This file contains all Prodigal ORF predictions for the long-read MQ bins. (GZ 18904 kb)

Additional file 14: Virus-host associations. A listing of all associations (Hi-C linkage or long-read alignment) between predicted viral contigs and non-viral contigs. (XLSX 24 kb)

Additional file 15: ARG allele predictions. A listing of all predicted candidate antibiotic resistance gene (ARG) alleles in the short- and long-read assemblies. (XLSX 17 kb)

Additional file 16: Long-read assembly ARG allele similarities. A percent identity matrix of detected ARG alleles to show high degrees of similarity between alleles. (XLSX 37 kb)

## Data Availability

The datasets generated and/or analyzed during the current study are available in the NCBI SRA repository under Bioproject: PRJNA507739 [58]. The assemblies [59, 60], bins [61–64], and ORF predictions [65, 66] are available on Figshare. A description of commands, scripts, and other materials used to analyze the data in this project can be found in the following GitHub repository: https://github.com/njdbickhart/RumenLongReadASM [67] and also on Zenodo [68].
