# Supplementary material for: Assignment of virus and antimicrobial resistance genes to microbial hosts in a complex microbial community by combined long-read assembly and proximity ligation
Source: Genome Biol. 2019 Aug 2;20:153. doi: 10.1186/s13059-019-1760-x (PMC6676630; doi:10.1186/s13059-019-1760-x)
Supplement: Supplementary file 1 — Supplementary figures and methods. Contains all supplementary figures and two supplementary tables. Additionally, contains a brief listing of additional methods and command line code for replicating analysis. (DOCX 3839 kb) [file 13059_2019_1760_MOESM1_ESM.docx]

**Supplementary Material for:**

**Assignment of virus and antimicrobial resistance genes to microbial hosts in a complex microbial community by combined long-read assembly and proximity ligation**

Derek M. Bickhart*^1^, Mick Watson*^2^, Sergey Koren*^3^, Kevin Panke-Buisse^1^, Laura M. Cersosimo^4^, Maximilian O. Press^5^, Curtis P. Van Tassell^6^, Jo Ann S. Van Kessel^7^, Bradd J. Haley^7^, Seon Woo Kim^7^, Cheryl Heiner^8^, Garret Suen^9^, Kiranmayee Bakshy^1^, Ivan Liachko^5^, Shawn T. Sullivan^5^, Phillip R. Myer^10^, Jay Ghurye^11^, Mihai Pop^11^, Paul J. Weimer^1,9^, Adam M. Phillippy^3^, Timothy P.L. Smith^12⸸^

1 Cell Wall Biology and Utilization Laboratory, Dairy Forage Research Center, USDA, Madison, WI, 53706, USA

2 Division of Genetics and Genomics, The Roslin Institute, Royal (Dick) School of Veterinary Studies, University of Edinburgh, Easter Bush, EH25 9RG, UK

3 Genome Informatics Section, Computational and Statistical Genomics Branch, National Human Genome Research Institute, Bethesda, Maryland, USA

4 Department of Animal Sciences, University of Florida, Gainesville, FL, 32611, USA

5 Phase Genomics Inc., Seattle, WA 98109, USA

6 Animal Genomics and Improvement Laboratory, Beltsville Agricultural Research Center, Agricultural Research Service, USDA, Beltsville, MD 20705 USA

7 Environmental Microbial and Food Safety Laboratory, Beltsville Agricultural Research Center, Agricultural Research Service, USDA, Beltsville, MD 20705 USA

8 Pacific Biosciences, Menlo Park, CA, USA

9 Department of Bacteriology, University of Wisconsin-Madison, Madison, WI, 53706, USA

10 Department of Animal Science, University of Tennessee, Knoxville, TN, 37996, USA

11 Department of Computer Science, University of Maryland, College Park, MD, 20742, USA

12 USDA-ARS U.S. Meat Animal Research Center, Clay Center, NE, 68933, USA

Table S1: Data sequenced for this survey

| Platform | Number of flowcells | Total cumulative bases |
| --- | --- | --- |
| Illumina (short-read) NextSeq 500 | 1 (four lanes) | 172 gigabases |
| Illumina (Hi-C) Hiseq2000 | 2 (libraries) | 10.1 gigabases |
| PacBio RSII | 8 | 6.7 gigabases |
| PacBio Sequel | 21 | 45.35 gigabases |

Table S2: Rumen short-read WGS datasets used in binning or comparisons in this study.

| SRA Accession | Total reads | Short-read mapping (%) | Long-read mapping (%) | Used in MetaBat binning? | Used in Hypergeometric enrichment test?^1^ |
| --- | --- | --- | --- | --- | --- |
| PRJEB10338 | 832,702,032 | 62 | 38 | Yes | Yes |
| PRJEB21624 | 5,756,832,251 | 70 | 47 | Yes | Yes |
| PRJEB8939 | 329,335,090 | 71 | 48 | Yes | Yes |
| PRJNA214227 | 1,571,755,419 | 61 | 41 | Yes | Yes |
| PRJNA291523 | 281,175,435 | 79 | 65 | Yes | Yes |
| PRJNA60251 | 2,647,283,000 | 36 | 20 | Yes | Yes |
| PRJNA255688 | 16,506,517 | 97 | 79 | Yes | No |
| PRJNA270714 | 67,813,340 | 66 | 35 | Yes | No |
| PRJNA280381 | 95,781 | 99 | 99 | Yes | No |
| PRJNA366460 | 44,383,305 | 82 | 57 | Yes | No |
| PRJNA366463 | 22,354,786 | 84 | 65 | Yes | No |
| PRJNA366471 | 23,525,510 | 82 | 63 | Yes | No |
| PRJNA366487 | 21,116,112 | 87 | 57 | Yes | No |
| PRJNA366591 | 45,294,312 | 81 | 63 | Yes | No |
| PRJNA366667 | 20,775,651 | 90 | 68 | Yes | No |
| PRJNA366681 | 22,410,058 | 84 | 61 | Yes | No |
| PRJNA398239 | 11,758,630 | 99 | 99 | Yes | No |
| PRJNA507739 (this study) | 1,148,721,358 | 85 | 47 | Yes | Yes |

^1^ Datasets that were comprised of over 100 million WGS short-reads were used in the enrichment test.

**Figure S1 – PacBio read alignments to Long-read assembly partitioned by GC%**

**
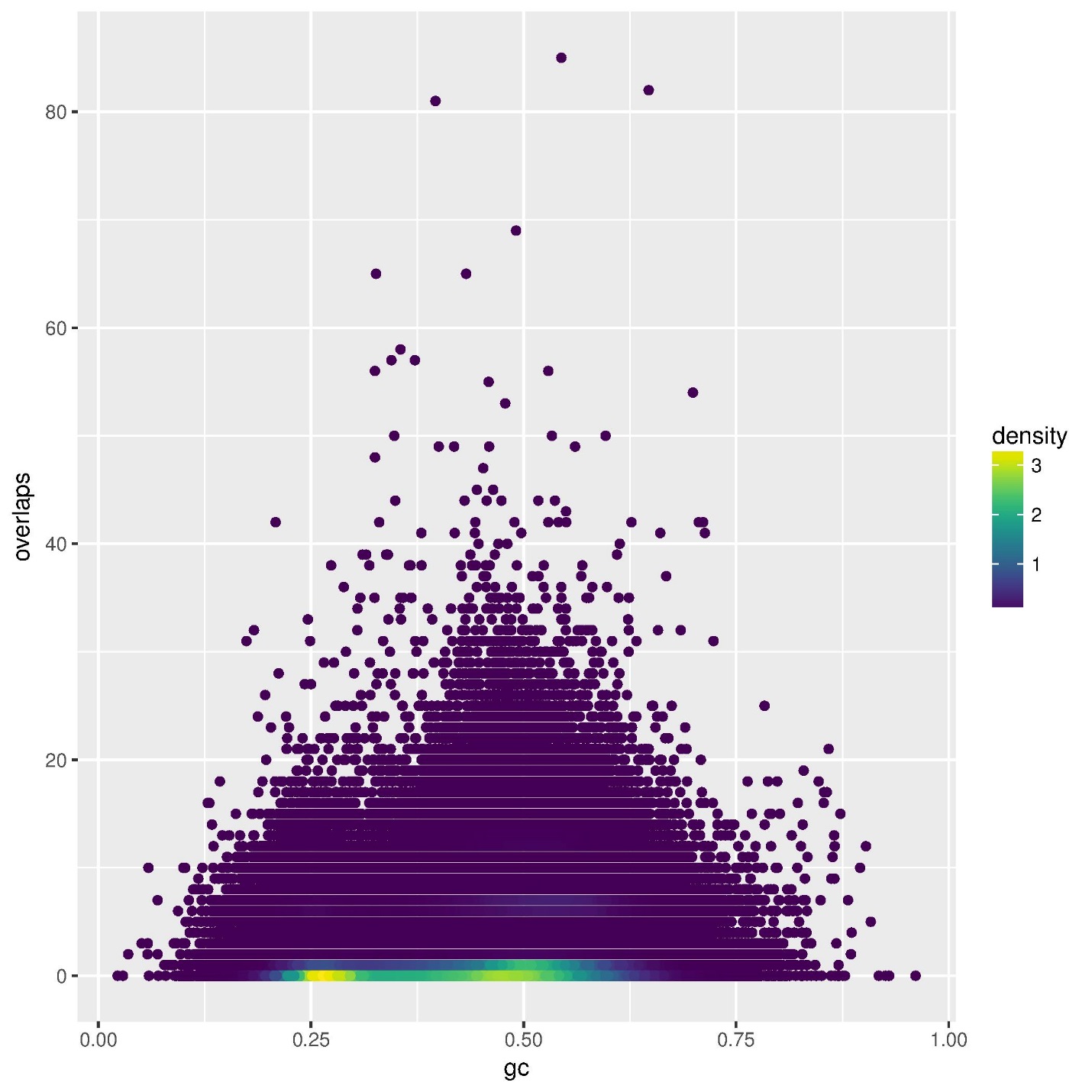
**

Density scatterplot showing the relationship of error-corrected long-read alignment counts (y-axis; “overlaps”) to the average GC% of long-read contigs (x-axis). Density increments are colored based on the number of overplotted points (increasing from dark blue to yellow).

**Figure S2 – Short-Read assembly contig length vs GC% confidence interval plot**

**
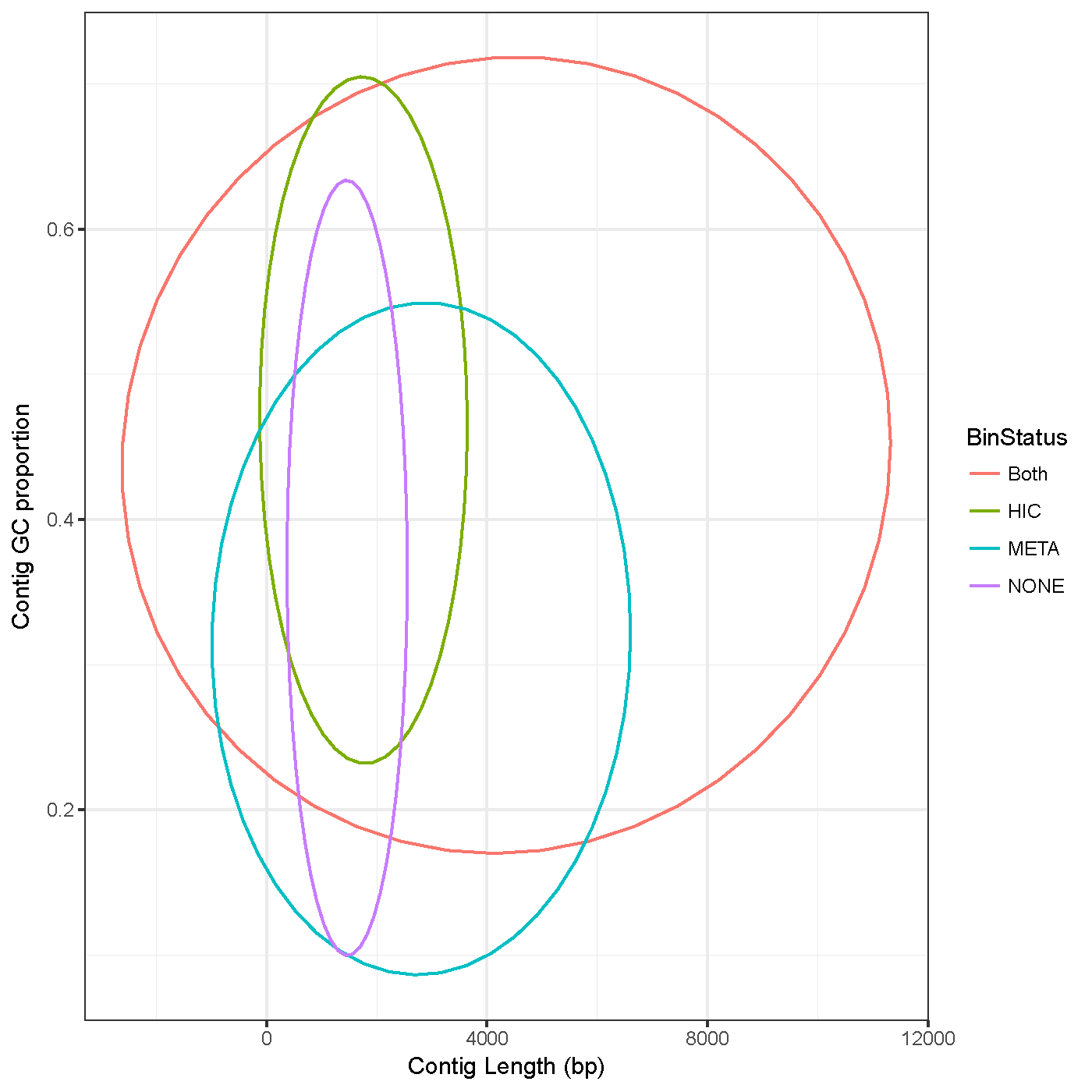
**

Confidence interval plots of short-read assembly contig lengths (x-axis) by average GC% (y-axis), by bin category (Both = Proximeta + Metabat binning; HIC = Proximeta binning only; META = Metabat binning only; NONE = Not used by either binning method). Confidence intervals represent the 95^th^ percentile for each bin category.

Figure S3 – Krona plot of short-read assembly contig taxonomic assignment


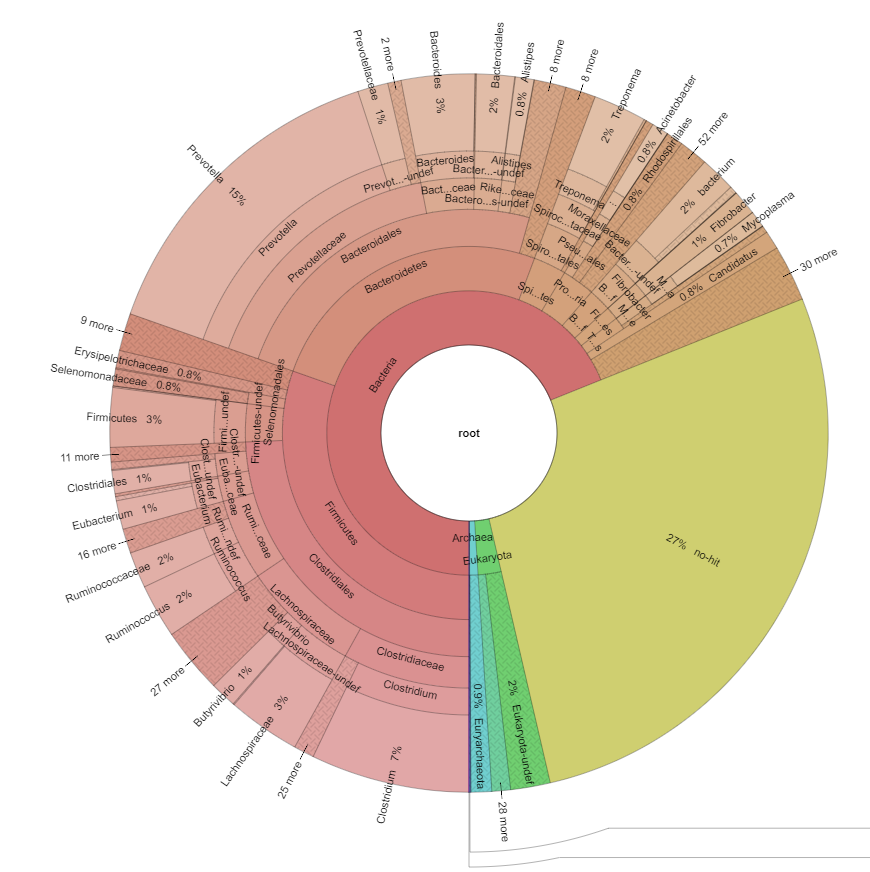


A Krona diagram showing the proportion of short-read assembly contigs assigned to distinct taxa. Taxonomic affiliations were identified using the BlobTools pipeline, with DIAMOND alignment (see methods for more details).

Figure S4 – Krona plot of long-read assembly contig taxonomic assignment


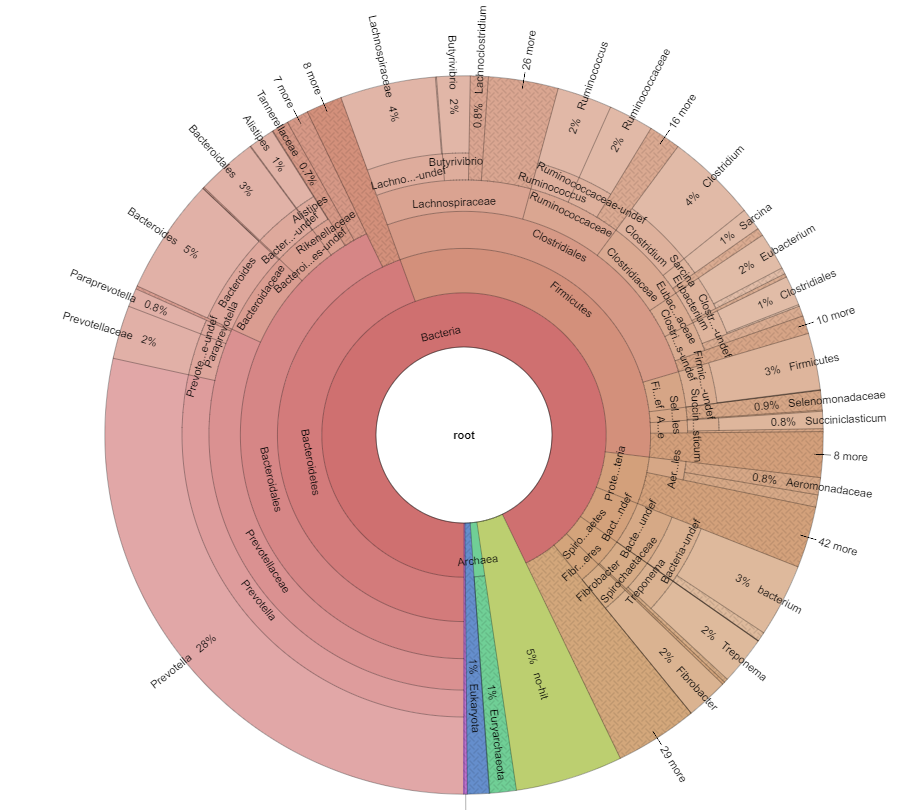


A Krona diagram showing the proportion of long-read assembly contigs assigned to distinct taxa. Taxonomic affiliations were identified using the BlobTools pipeline (1), with DIAMOND alignment (see methods for more details).

Figure S5 – Hi-C intercontig link association of ARG allele-containing and other contigs


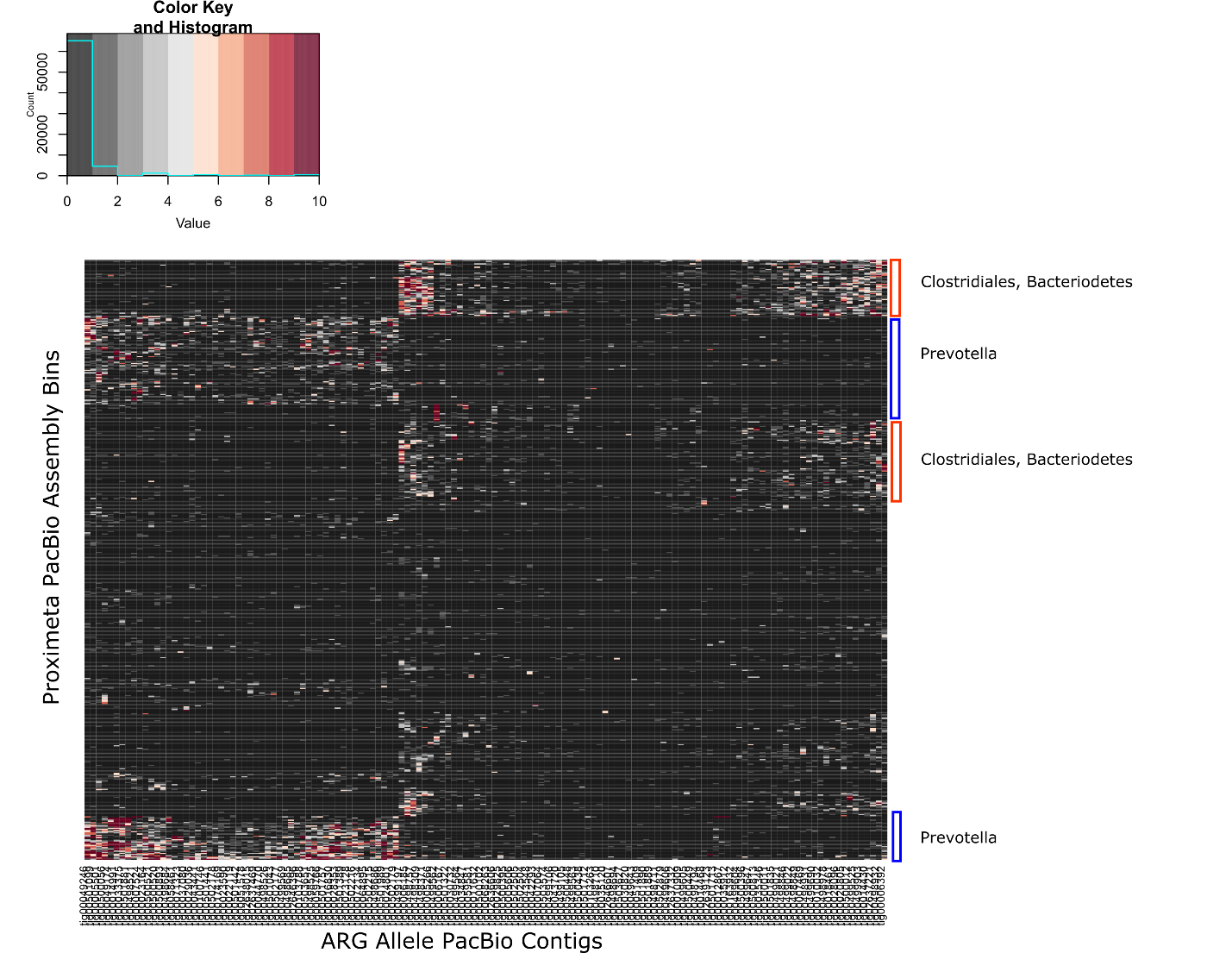


A heatmap showing the number of Hi-C intercontig links (an integer count of the number of mappings) between ARG allele-containing contigs (x-axis) and contigs from different bins (y-axis). Contig groupings on the y-axis were labelled if they belonged to consistent taxonomic genera (ie. Prevotella).

Figure S6 – short-read assembly differential coverage plot

**
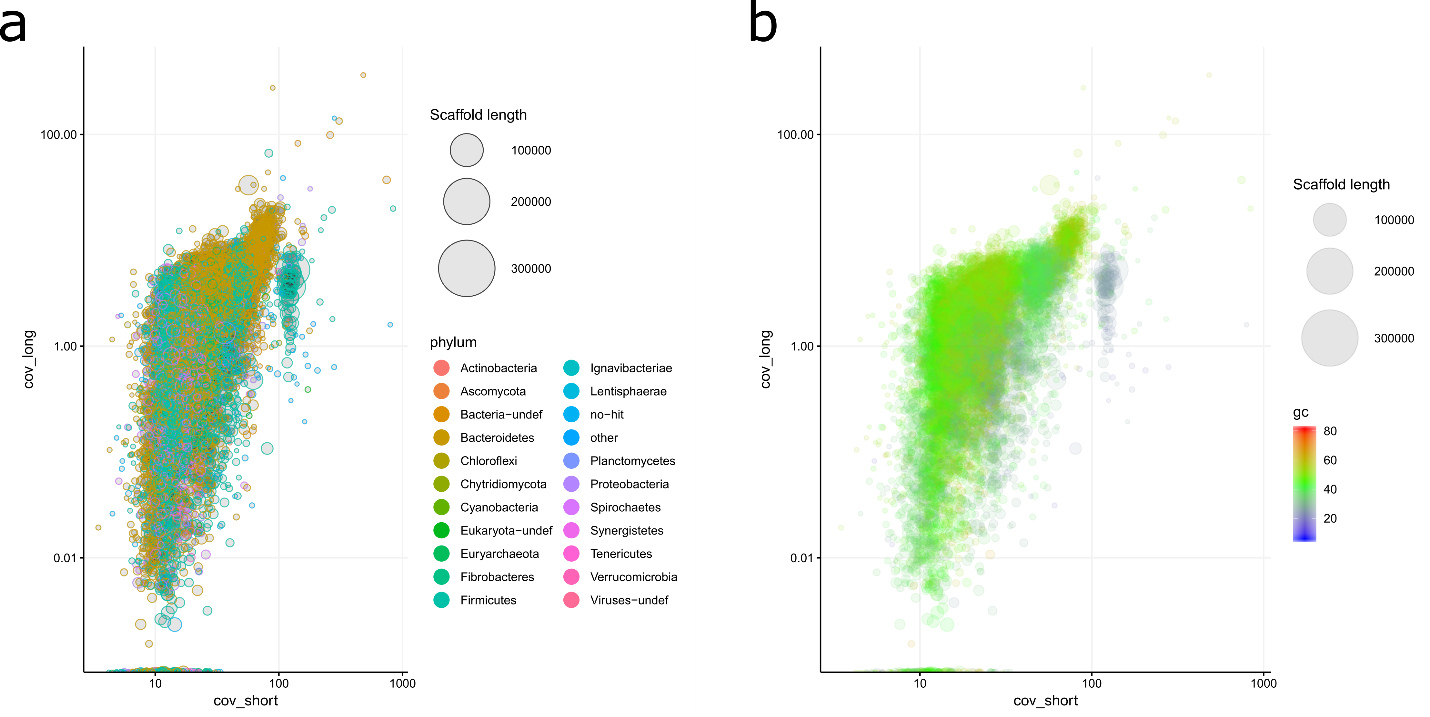
**

Differential coverage plots generated with mmgenome2 (2) on the short-read assembly HQ bin contigs. Both plots compare the coverage of subsampled long-reads (y axis) with short-reads (x axis) used to generate the assemblies in this manuscript. Contigs are colored by (a) Blobtools (1) taxonomic affiliation or (b) the average GC% of the contig.

Figure S7 – long-read assembly differential coverage plot

**
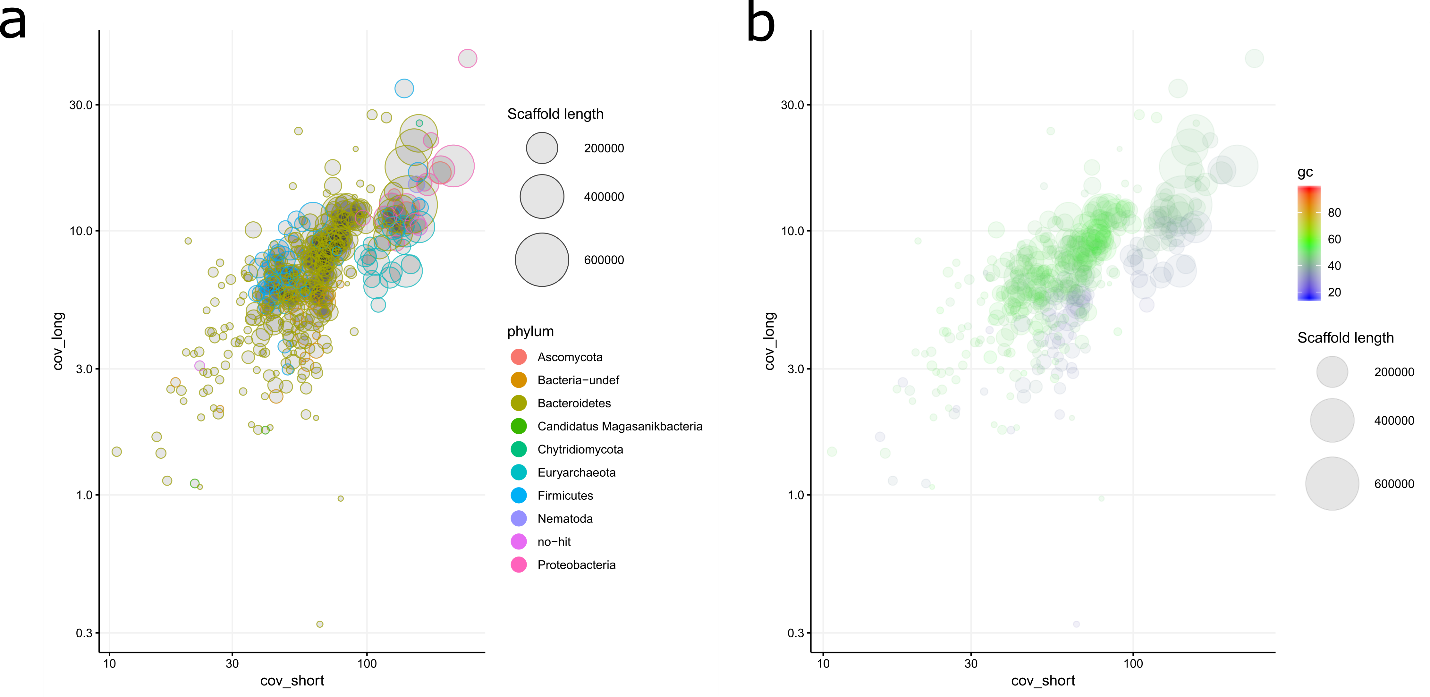
**

Differential coverage plots generated with mmgenome2 (2) on the long-read assembly HQ bin contigs. Both plots compare the coverage of subsampled long-reads (y axis) with short-reads (x axis) used to generate the assemblies in this manuscript. Contigs are colored by (a) Blobtools (1) taxonomic affiliation or (b) the average GC% of the contig.

Figure S8 – Fragment analyzer spectra for rumen contents sample prepared via modified Yu and Morrison protocol.

Fragment analyzer spectra showing the average DNA fragment size achieved from the extraction protocol. DNA fragment size reached an average of 14 kbp; however, sequence subread lengths were shorter, suggesting that single-strand nicks occurred during the extraction.

Figure S9 – short-read assembly, Hi-C vs short-read differential coverage plot


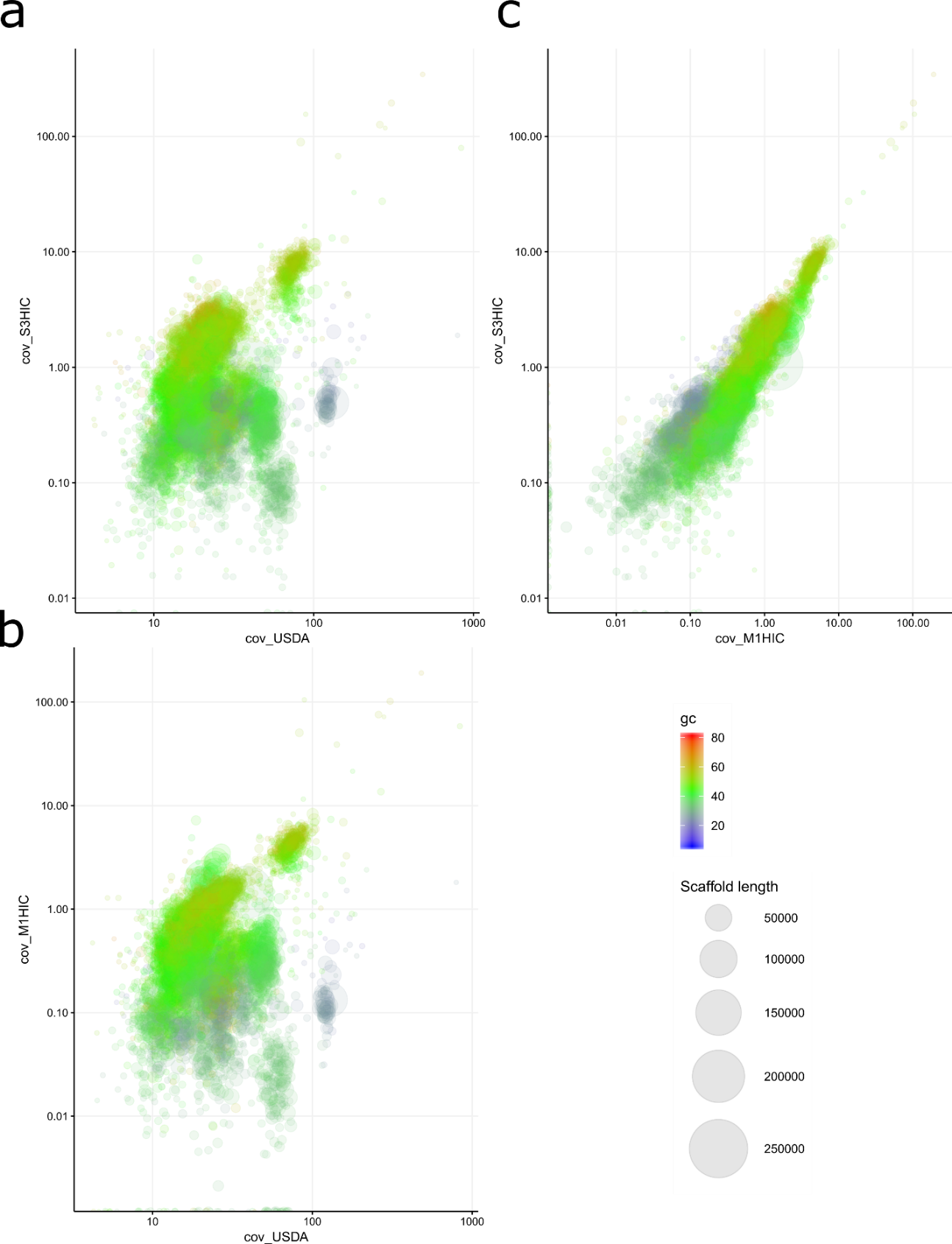


Differential coverage plots showing the log10 coverage of the short-read assembly (x-axis) vs the Hi-C S3 (a) and Hi-C M1 (b) libraries. A comparison of the coverage profile of the Hi-C reads against each other (c) revealed little coverage bias between the two libraries.

Figure S10 – long-read assembly, Hi-C vs short-read differential coverage plot


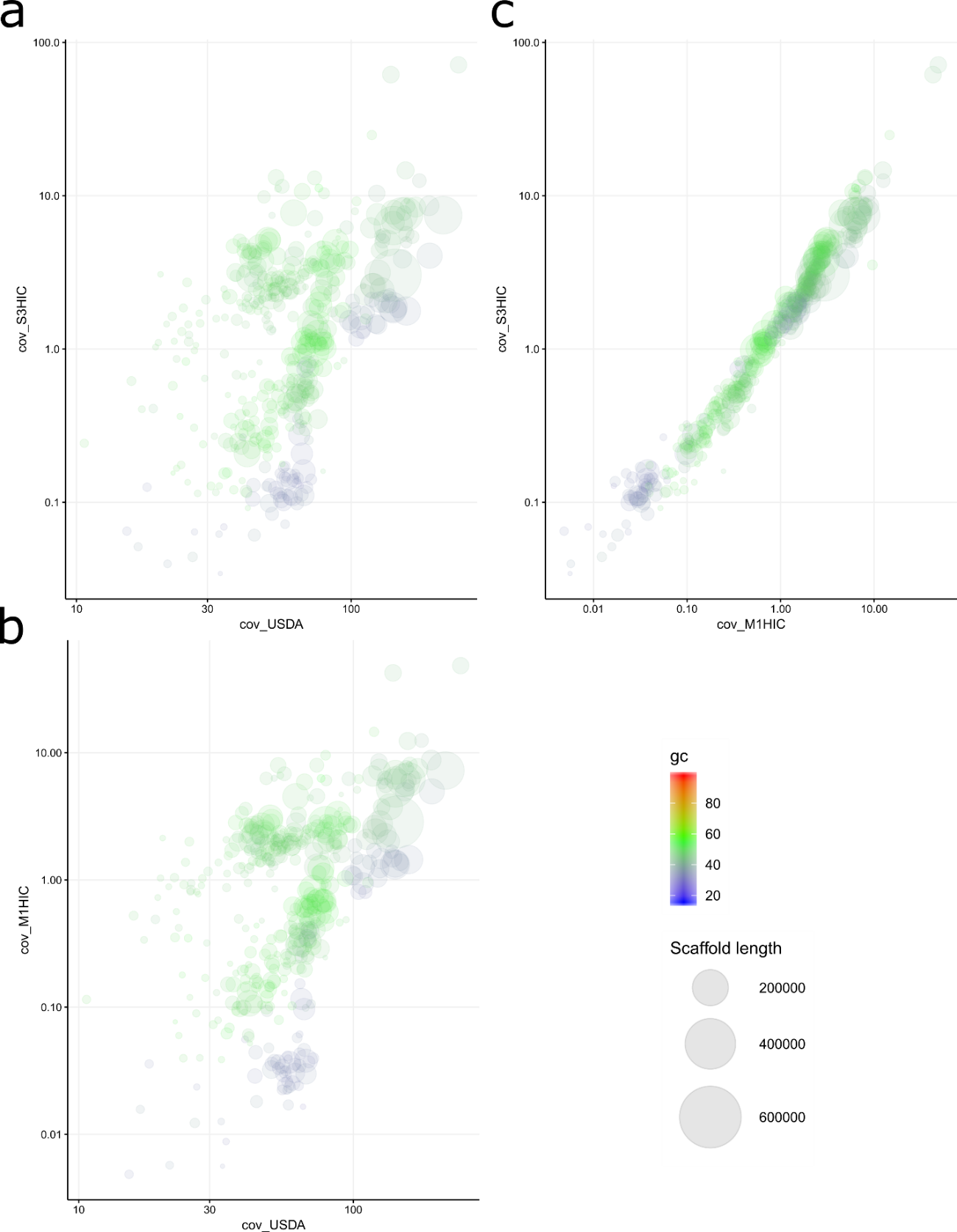


Differential coverage plots showing the log10 coverage of the long-read assembly (x-axis) vs the Hi-C S3 (a) and Hi-C M1 (b) libraries. A comparison of the coverage profile of the Hi-C reads against each other (c) revealed little coverage bias between the two libraries on this assembly.

Figure S11 – Hi-C read mapping position proximity to restriction site motif


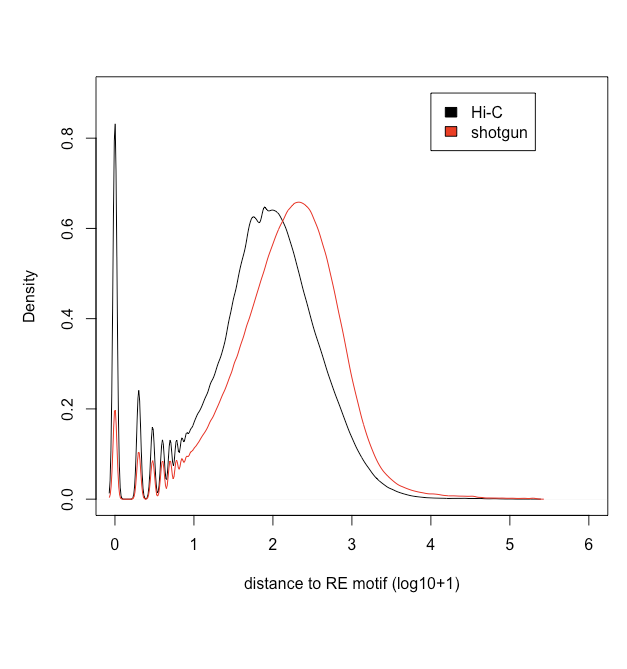


Plot of the distance of read mappings from MluCI and Sau3AI restriction sites for the short-read WGS paired end library (red) and the Hi-C reads themselves (black). Hi-C reads tended to have a closer association with restriction sites than raw WGS reads, as expected.

Figure S12 – short-read WGS alignment percentages to assembled bins


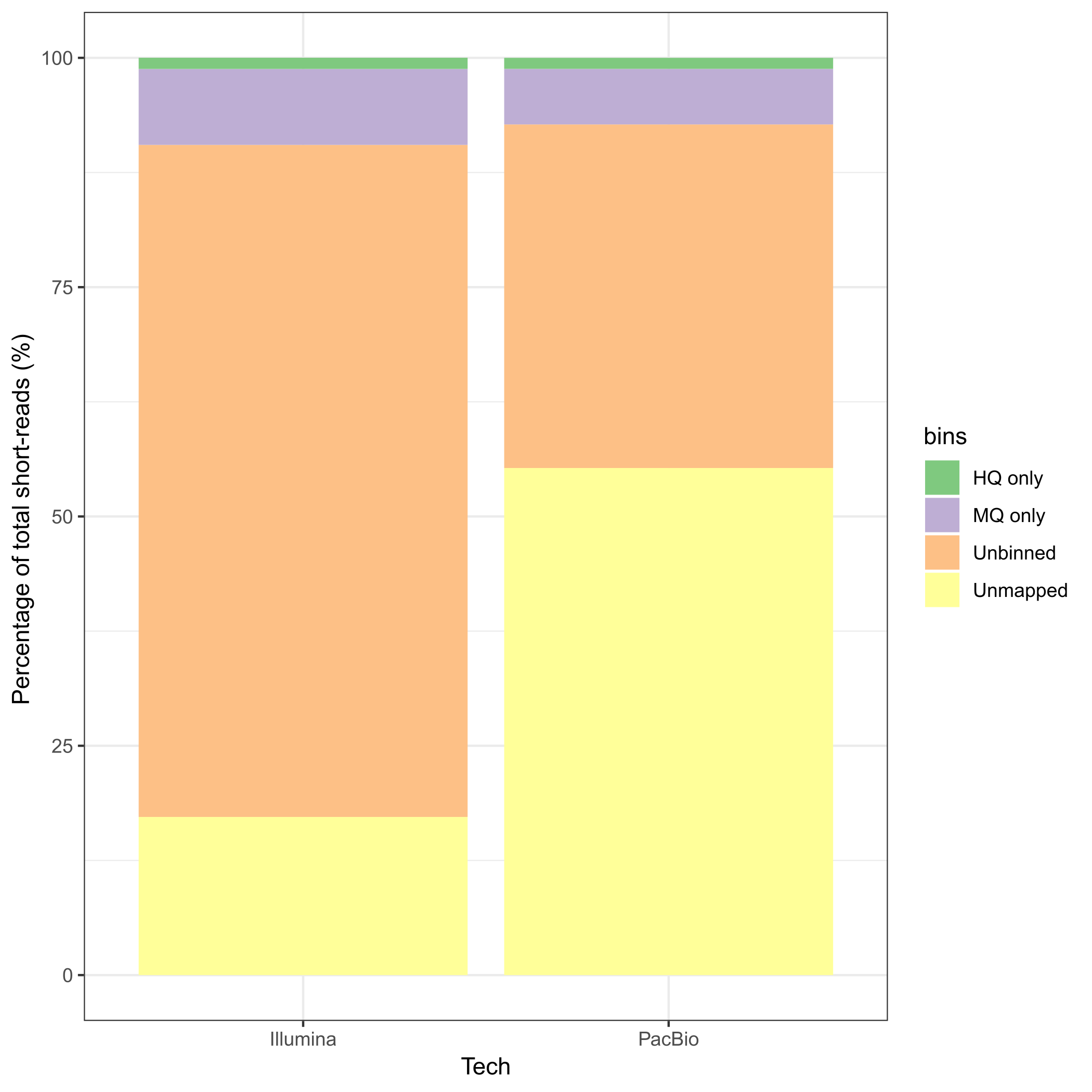


Comparative mapping proportions of short-read WGS data to binned and unbinned portions of the short-read (Illumina) and long-read (PacBio) datasets. The WGS data (the same data used to generate the short-read assembly) was remapped to each assembly and proportions that aligned to contigs that were binned in the HQ bins, MQ bins and unbinned contigs were counted. Short-reads that did not map to contigs in the assembly were counted as “Unmapped.”

Figure S13 – short-read assembly CheckM bin statistics


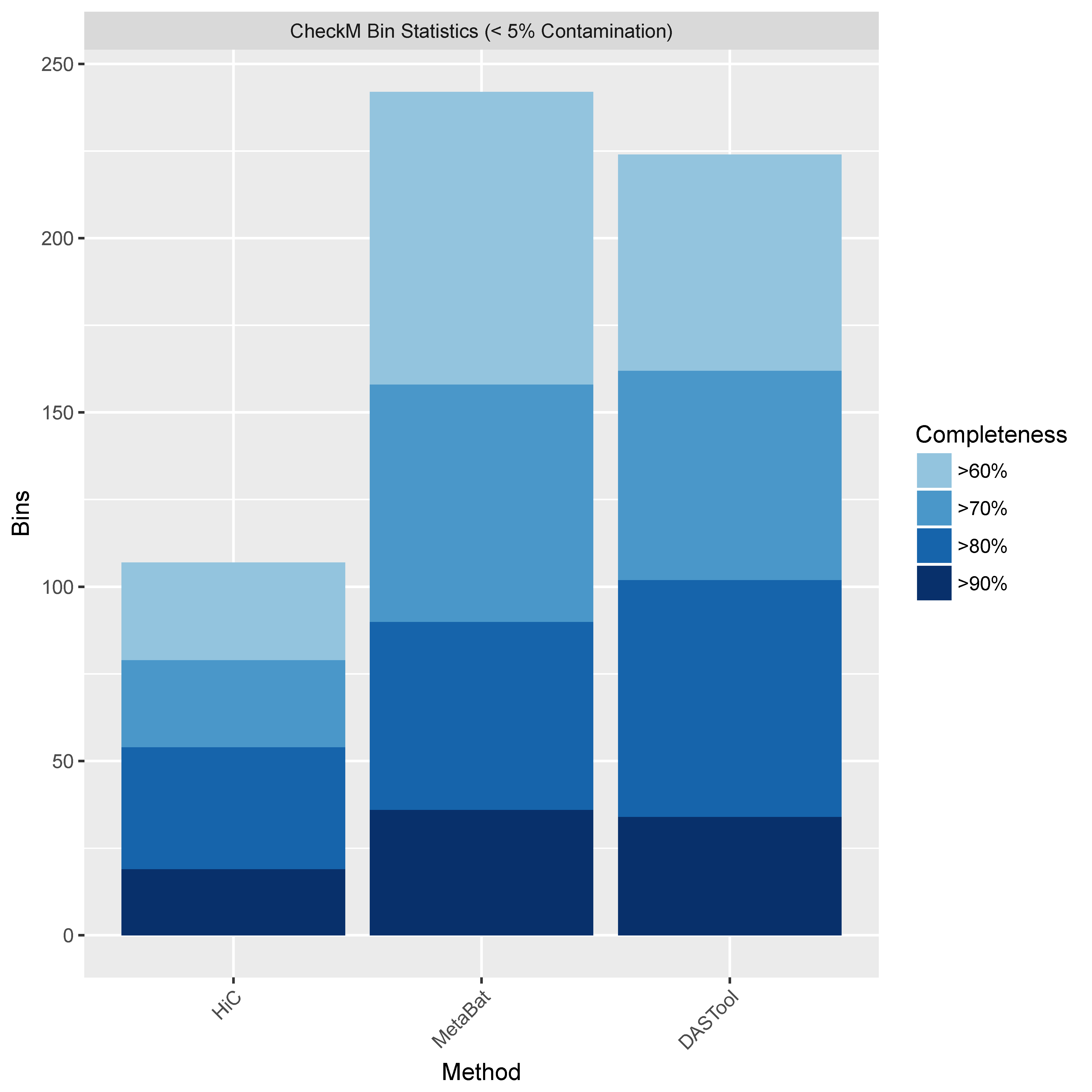


Comparative bin quality statistics of bins from the short-read assembly as assessed by the CheckM (3) tool. Bin counts (y-axis) only consist of bins with less than 5% contamination at each level of completeness.

Figure S14 – Long-read assembly CheckM bin statistics


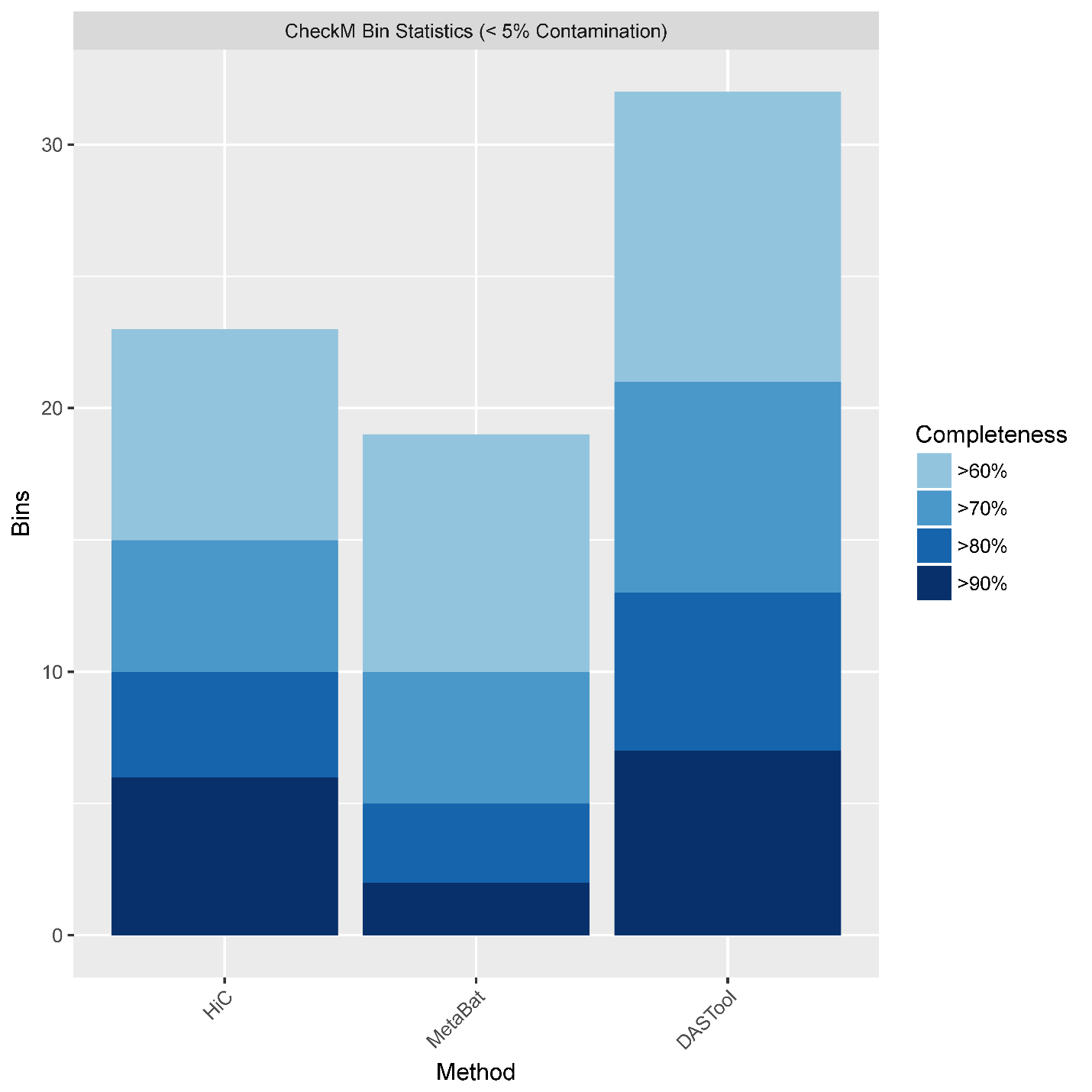


Comparative bin quality statistics of bins from the long-read assembly as assessed by the CheckM (3) tool. Bin counts (y-axis) only consist of bins with less than 5% contamination at each level of completeness.

**Supplementary methods**

**Binning strategies**

In order to associate disparate contigs into biologically relevant bins, we used one computational and one technical method. We used MetaBat2(4)(referred to as “MetaBat” hereafter) to group contigs by tetranucleotide frequency and sequence coverage metrics. Sequence coverage was generated for each assembly by aligning paired-end short reads from our input dataset and 16 public SRA datasets (Table S17) using BWA MEM(5) and Samtools(6). Coverage statistics were generated from each alignment BAM file via the use of the jgi_summarize_bam_contig_depths executable provided in the MetaBat2 distribution, using default settings and the “—outputDepth” argument. MetaBat2 was run with the combined coverage statistics data on the short-read and long-read assemblies, respectively. Contig-bin assignments were tabulated from the resulting MetaBat fasta files using this custom script:

perl -e '@f = `ls public_metabat/*.fa`; chomp(@f); foreach $h (@f){@hsegs = split(/\./, $h); open(IN, "< $h"); while(<IN>){if($_ =~ /^>/){chomp; $_ =~ s/>//; print "$_\t$hsegs[-2]\n";}} close IN;}' > bin_ids.tab

Hi-C bins were generated by Phase Genomics (Seattle, WA) using their ProxiMeta analysis service. Briefly, this technique uses proximity ligation (Hi-C) connectivity between contigs to group contigs into bins. For more details about ProxiMeta clustering and analysis see (7). Similar to the analysis in Press et al. 2017, we prepared two Hi-C libraries with the MluCI and Sau3AI restriction enzymes, respectively (4). For the MluCI Hi-C library, 24% of read pairs map to different contigs than their mate, whereas for the Sau3AI Hi-C library 25% of read pairs map to different contigs than their mate. This is slightly less than half of the total reads pairs that map in each case. By contrast, only 14% of read pairs from the short-read WGS data used to generate the short-read assembly map to two different contigs. To assess Hi-C library quality, we sought to compare the distribution of read mappings around expected restriction endonuclease (RE) motifs. Hi-C read mapping distance to RE motifs can be thought of as sampling a RE motif and then sampling an insert around the motif, the ends of which constitute the reads. Thus, in some cases with rather short inserts or rather long reads, the RE junctions will often be present in the reads. To show this, we examined the distribution of distances between read mappings (both Hi-C and shotgun) and RE motifs. We used the script fastaRegexFinder.py v0.1.0 (https://github.com/dariober/bioinformatics-cafe/blob/master/fastaRegexFinder/fastaRegexFinder.py) to annotate the PacBio assembly with RE motifs. We then used BEDTools v2.26.0 to convert Hi-C mappings to BED format and investigate the distances of mappings to motifs, using the BEDtools sub-command “closest”, and using only the leftmost coordinate of each read (to account for different read lengths between shotgun and Hi-C reads). It is important to note that the libraries used in this study have an expected insert size of approximately 500-600 bp, so most reads are not expected to contain the RE motif. In Figure S11 we show the distribution of such distances for both shotgun reads and Hi-C reads (note log10 scale). In this case we observe that Hi-C reads are on average 254.3 bp away from a motif, whereas shotgun reads are 614.7 bp away from a motif (Kolmogorov-Smirnov test: D = 0.2, p < 2*10-16). These observations comport with the expected 500-600bp insert size of the Hi-C library (each read’s start will be ~250-300bp from the midpoint of the insert, which is the average location of a RE junction).

We then used the DAS_Tool pipeline (8) to consolidate the MetaBat and Hi-C bins and to assess bin quality using single-copy-gene (SCG) annotations. DAS_Tool was run with Diamond(9) as the default search engine and used the same Prodigal (10) ORF predictions identified through the BlobTools (9) pipeline to identify SCGs. We also ran DAS_Tool without a minimum reporting bin_score threshold so that statistics could be generated on each consolidated binset:

DAS_Tool -i pacbio_final_public_hic.unsorted.bins,pacbio_final_public_metabat.unsorted.bins -c usda_pacbio_second_pilon_indelsonly.fa -o pacbio_final_dastool -l HiC,metabat --search_engine diamond -t 10 --db_directory /mnt/nfs/nfs2/bickhart-users/binaries/DAS_Tool/db --write_bins 1 --proteins pacbio_final_prodigal_proteins.faa --score_threshold 0

DAS_Tool -i illumina_megahit_hic.unsorted.bins,illumina_megahit_public_metabat.unsorted.bins -c mick_megahit_final_full.rfmt.fa -o illumina_megahit_dastool -l HiC,metabat --search_engine diamond -t 10 --db_directory /mnt/nfs/nfs2/bickhart-users/binaries/DAS_Tool/db --write_bins 1 --proteins illumina_megahit_prodigal_proteins.faa --score_threshold 0

**Hybrid genome assembly**

As we had substantially greater depth of coverage with the short-read dataset, we attempted to scaffold our short-read assembly contigs with our long-read data using the Opera-LG scaffolder (11). Due to the constraints placed on Opera-LG by its optimized data structures, we were unable to use it to scaffold the entire short-read assembly as that assembly was larger than the 4 gigabase limit on input assemblies. To circumvent this limitation, we removed all contigs less than 1.5 kb from the short-read assembly before scaffolding. We observed only a minor improvement in scaffold N50 length and a substantial increase in N-base gap content in the hybrid assembly. Despite the longer read lengths in our long-read dataset, there was insufficient overlap and coverage on the short-read assembly contigs to generate substantially longer scaffolds.

**PacBio alignment and Hi-C inter-cluster link analysis for Viral host-specificity analysis**

Error corrected read alignments of PacBio reads were also used to identify and confirm viral host-specificity in assembled contigs from both assemblies. Filtered alignments of long-reads that spanned BlobTools/DIAMOND designated viral contigs and non-viral contigs resulted in a list of 64 and 45 candidate insertions of long-read and short-read viral contigs, respectively, in other non-viral contigs. In order to identify these associations, we first filtered read alignments of error-corrected PacBio reads to an exclusive reference set of viral contigs and identified the segments of the reads that were overhanging the end of the alignment:

minimap2 -x map-pb pacbio_pilon_viruses.fa rumen_pacbio_corrected.fasta.gz > pacbio_pilon_viruses_ecpbreads.paf

perl selectLikelyViralOverhangs.pl pacbio_pilon_viruses_ecpbreads.paf pacbio_pilon_viruses_ecpbreads.filt

We then kept all read overhangs that were greater than 150 bp in length, and extracted the portion of the error corrected reads that were previously unmapped to the viral contigs:

perl -lane 'if($F[2] - $F[1] > 150){print $_;}' < pacbio_pilon_viruses_ecpbreads.filt.subread.bed > pacbio_pilon_viruses_ecpbreads.filt.subread.gt150.bed

perl -e '@list; while(<>){chomp; @s = split(/\t/); push(@list, "$s[0]:$s[1]-$s[2]"); if(scalar(@list) > 500){print "Printing...\n"; system("samtools faidx ../../sequence_data/pilot_project/pacbio/rumen_pacbio_corrected.fasta " . join(" ", @list) . " >> pacbio_pilon_viruses_ecpbreads.filt.subread.gt150.fa"); @list = ();}} system("samtools faidx ../../sequence_data/pilot_project/pacbio/rumen_pacbio_corrected.fasta " . join(" ", @list) . " >> pacbio_pilon_viruses_ecpbreads.filt.subread.gt150.fa");' < pacbio_pilon_viruses_ecpbreads.filt.subread.gt150.bed

The overhanging read data was realigned to the entire assembly and Blobtools-derived taxonomic information was used to filter and label the association data.

perl -e 'chomp(@ARGV); open(IN, "< $ARGV[0]"); %data; while(<IN>){chomp; @s = split(/\t/); if($s[11] == 0){next;} $s[0] =~ s/\:\d+\-\d+$//; push(@{$data{$s[0]}}, $s[5]);} close IN; open(IN, "< $ARGV[1]"); while(<IN>){chomp; @s = split(/\t/); if(exists($data{$s[0]})){push(@{$data{$s[0]}}, $s[5]);}} close IN; foreach my $k (keys(%data)){print "$k\t" . join("\t", @{$data{$k}}) . "\n";}' pacbio_pilon_viruses_ecpbreads.filt.subread.gt150.paf pacbio_pilon_viruses_ecpbreads.paf > pacbio_pilon_viruses_ecpbreads.assoc.filt.tab

perl generateViralAssociationGraph.pl pacbio_secpilon_blobplot_all.pacbio_secpilon_blobplot.blobDB.table.txt pacbio_pilon_viruses_ecpbreads.assoc.filt.stringent.tab pacbio_pilon_viruses_ecpbreads.assoc.filt.stringent.cyto.tab

In order to identify Hi-C inter-cluster links, we first generated a bipartite inter-contig Hi-C read graph by aligning Hi-C read pairs to each assembly and by selecting alignments where one read pair aligned to a viral contig and the other aligned to a non-viral contig. We then created a sum value for the cardinal counts of each unique association, and filtered the resulting data by the magnitude of the count. We chose values of 10 and 20 observations as filter cutoffs for the short-read and long-read viral-host graphs, respectively, as those values represented the median counts for each dataset. This filtered data was used to generate an extended table file similar to the PacBio read alignment data listed above:

perl generateViralAssociationGraph.hiclinks.pl ../blobtools/illumina_blobplot_all.illumina_megahit_blobplot.blobDB.table.txt illumina_viruses.hiclinks.filt.tab illumina_viruses.hiclinks.filt.cyto.tab

Finally, the Hi-C inter-cluster links and PacBio read alignments were combined into a final association file using a simple overlap of the viral-host associations discovered in each separate analysis.

perl combineViralSignal.pl pacbio_pilon_viruses.hiclinks.filt.cyto.tab pacbio_pilon_viruses_ecpbreads.stringent.cyto.tab > pacbio_pilon_viruses.combined.cyto.tab

The resulting table was loaded into Cytoscape version 3.6.1 (12), with the viral contigs and non-viral contigs selected as nodes, and the method of detection {“PacBio”, “HIC”, “BOTH”} selected as the edge/interaction. In the case of the long-read assembly data, viral nodes that had more than two connections with non-viral nodes were grouped using an “Attribute circle” layout. The short-read assembly had fewer of these high connectivity nodes, so the default network layout was used to display the network.

We have adapted many of the features of this workflow into a formal pipeline on the associated github repository for this manuscript. Details on how to replicate this analysis and the source code for the scripts can be found at this URL: <https://github.com/njdbickhart/RumenLongReadASM/tree/master/viralAnalysisScripts>

**Hypergeometric determination of contig coverage enrichment**

Short-read sequence data alignments from six of the 16 SRA datasets used to bin the contigs (see Table S17) were used in the subsequent analysis of coverage enrichment. In each test, we calculated the proportion of the total aligned reads from each respective dataset to each contig from the short-read and long-read MQ bins. We grouped contigs by their likely genus origin based on their Blobtools taxonomic designation. We then identified cases where our short-read dataset had the most or the least aligned reads to each contig and classified them as “max” or “min” events, respectively. Grouping contigs by their genus designation, we then tested whether the proportion of “max” or “min” read depth contigs observed were higher than expected in the group using the Scipy hypergeometric survivor function (scipy.stats.hypergeom.sf). To correct for multiple hypothesis testing, we applied a Benjamini-Hochberg correction to the hypergeometric survivor function p values using an alpha of 0.05.

**References**

1. Laetsch DR, Blaxter ML. BlobTools: Interrogation of genome assemblies. F1000Research. 2017 Jul 31;6:1287.

2. Karst SM, Kirkegaard RH, Albertsen M. mmgenome: a toolbox for reproducible genome extraction from metagenomes. bioRxiv. 2016 Jun 15;059121.

3. Parks DH, Imelfort M, Skennerton CT, Hugenholtz P, Tyson GW. CheckM: assessing the quality of microbial genomes recovered from isolates, single cells, and metagenomes. Genome Res. 2015 Jul;25(7):1043–55.

4. Kang DD, Froula J, Egan R, Wang Z. MetaBAT, an efficient tool for accurately reconstructing single genomes from complex microbial communities. PeerJ [Internet]. 2015 Aug 27 [cited 2017 Apr 10];3. Available from: http://www.ncbi.nlm.nih.gov/pmc/articles/PMC4556158/

5. Li H, Durbin R. Fast and accurate short read alignment with Burrows-Wheeler transform. Bioinforma Oxf Engl. 2009 Jul 15;25(14):1754–60.

6. Li H, Handsaker B, Wysoker A, Fennell T, Ruan J, Homer N, et al. The Sequence Alignment/Map format and SAMtools. Bioinforma Oxf Engl. 2009 Aug 15;25(16):2078–9.

7. Press MO, Wiser AH, Kronenberg ZN, Langford KW, Shakya M, Lo C-C, et al. Hi-C deconvolution of a human gut microbiome yields high-quality draft genomes and reveals plasmid-genome interactions. bioRxiv. 2017 Oct 5;198713.

8. Sieber CMK, Probst AJ, Sharrar A, Thomas BC, Hess M, Tringe SG, et al. Recovery of genomes from metagenomes via a dereplication, aggregation and scoring strategy. Nat Microbiol. 2018 Jul;3(7):836–43.

9. Buchfink B, Xie C, Huson DH. Fast and sensitive protein alignment using DIAMOND. Nat Methods. 2015 Jan;12(1):59–60.

10. Hyatt D, Chen G-L, LoCascio PF, Land ML, Larimer FW, Hauser LJ. Prodigal: prokaryotic gene recognition and translation initiation site identification. BMC Bioinformatics. 2010 Mar 8;11:119.

11. Gao S, Bertrand D, Chia BKH, Nagarajan N. OPERA-LG: efficient and exact scaffolding of large, repeat-rich eukaryotic genomes with performance guarantees. Genome Biol. 2016 May 11;17(1):102.

12. Shannon P, Markiel A, Ozier O, Baliga NS, Wang JT, Ramage D, et al. Cytoscape: A Software Environment for Integrated Models of Biomolecular Interaction Networks. Genome Res. 2003 Nov;13(11):2498–504.
